# Quantifying biodiversity trade-offs in the face of widespread renewable and unconventional energy development

**DOI:** 10.1038/s41598-020-64501-7

**Published:** 2020-05-05

**Authors:** Viorel D. Popescu, Robin G. Munshaw, Nancy Shackelford, Federico Montesino Pouzols, Evgenia Dubman, Pascale Gibeau, Matt Horne, Atte Moilanen, Wendy J. Palen

**Affiliations:** 1 Department of Biological Sciences and Sustainability Studies Theme, Ohio University, 107 Irvine Hall, Athens, OH 45701 USA; 2 Earth to Ocean Research Group, Department of Biological Sciences, Simon Fraser University, 8888 University Dr., Burnaby, BC V5A 1S6 Canada; 3 Centre for Environmental Research (CCMESI), University of Bucharest, 1 N. Balcescu Blvd, Bucharest, Romania; 4 Ecology and Evolutionary Biology, University of Colorado Boulder, 4100 Discovery Dr, Boulder Colorado, 80303 USA; 5 European Southern Observatory, Science Operation Software Department, Garching bei München, Germany; 6 City of Vancouver, Climate Policy, British Columbia, Canada; 7 Department of Geography and Geosciences, P.O. Box 64, FI-00014 University of Helsinki, Helsinki, Finland; 8 Finnish Natural History Museum, P.O. Box 17, FI-00014 University of Helsinki, Helsinki, Finland

**Keywords:** Environmental impact, Biodiversity, Conservation biology, Ecological modelling, Climate change, Energy infrastructure

## Abstract

The challenge of balancing biodiversity protection with economic growth is epitomized by the development of renewable and unconventional energy, whose adoption is aimed at stemming the impacts of global climate change, yet has outpaced our understanding of biodiversity impacts. We evaluated the potential conflict between biodiversity protection and future electricity generation from renewable (wind farms, run-of-river hydro) and non-renewable (shale gas) sources in British Columbia (BC), Canada using three metrics: greenhouse gas (GHG) emissions, electricity cost, and overlap between future development and conservation priorities for several fish and wildlife groups - small-bodied vertebrates, large mammals, freshwater fish – and undisturbed landscapes. Sharp trade-offs in global versus regional biodiversity conservation exist for all energy technologies, and in BC they are currently smallest for wind energy: low GHG emissions, low-moderate overlap with top conservation priorities, and competitive energy cost. GHG emissions from shale gas are 1000 times higher than those from renewable sources, and run-of-river hydro has high overlap with conservation priorities for small-bodied vertebrates. When all species groups were considered simultaneously, run-of-river hydro had moderate overlap (0.56), while shale gas and onshore wind had low overlap with top conservation priorities (0.23 and 0.24, respectively). The unintended cost of distributed energy sources for regional biodiversity suggest that trade-offs based on more diverse metrics must be incorporated into energy planning.

## Introduction

Biodiversity is declining at an alarming rate as a result of habitat loss, overexploitation, and climate change^1,2^. To address this challenge, 196 countries have signed the Convention on Biological Diversity, which aims to halt biodiversity loss by 2020 by reducing direct harm, increasing protected areas, mitigating climate, and reducing global carbon emissions. With global energy demand projected to increase by 14–33% by the year 2035^3^, these commitments have contributed to an exponential increase in the development of renewable electricity sources (e.g., wind, solar, biomass, hydropower^4^). Concomitantly, unconventional fossil fuels such as shale gas, are being developed worldwide (International Energy Agency; *iea.org*), and presented as less GHG intensive alternatives to coal-fired electricity generation^5^. The development of new renewable electricity has been dominated by distributed energy resources (e.g. wind, solar, small hydropower) that are assumed to be more environmentally benign than traditional, large scale technologies, such as large dams or coal-fired plants^6^. For example, the widespread adoption of renewable energy has resulted in spatially distributed interconnected networks of facilities that can range from small (i.e., single wind turbines or small hydropower dams, <5 or 10 MW installed capacity) to large wind and solar farms^7,8^.

Distributed energy resources pose interesting challenges to land use planning when the goal is to solve a grid-based energy problem^9^. For any given resource type, individual distributed energy resource facilities may have small physical footprints compared to single large facilities, but cumulatively may require substantially more infrastructure (roads, powerlines) per unit of energy produced (e.g., many small hydropower projects with little to no water storage capacity vs. a single large dam flooding vast areas)^10,11^. Similarly, shale gas extraction and transportation is characterized by a dense network of well pads, access roads, seismic lines, and pipelines which can cumulatively affect large geographic areas^12,13^. As such, both renewable distributed energy resources and shale development may result in similar spatial footprints that can be difficult to evaluate or integrate into land use planning, as well as conservation planning.

Given the rapid increase in renewable energy and shale gas development worldwide, there is an increasing need to understand the potential for cumulative environmental and biodiversity impacts of such technologies^14–16^. Policy-makers and ecologists alike recognize the need for strategic planning for renewable energy and unconventional fossil fuel development to navigate the complex trade-offs between reducing global GHG emissions, minimizing local impacts to biodiversity and human health, while meeting growing energy demands^17–21^. Thus, identifying optimal energy portfolios that meet economic, environmental and social constraints requires not only a stakeholder-engaged process that addresses social acceptance of emerging technologies^22,23^, but also the development of analytical frameworks that integrate multiple targets and consider the cumulative effects of energy development^24–27^. While many studies have focused on identifying optimal sites of renewable energy resources based on physical attributes of their target area and technical specifications of a particular technology, only few attempted to expand the optimization criteria to biotic, economic, or social factors^28–31^.

Renewable and unconventional energy development in North America has been characterized by an exponential increase in electricity generating capacity from wind and solar power (15-fold and 24-fold increase since 2000, respectively; US Department of Energy, *energy.gov*), as well as extraction of natural gas from shale (7-fold increase in gas production since 2007^14^). Until recently, the Province of British Columbia (BC), Canada, was undergoing a shift from large energy production infrastructure to a system of numerous small dispersed electricity production facilities installed and operated by private corporations^32^; though this focus has changed recently with the approval of a large dam on the Peace River (Site C; 1100 MW installed capacity capable of producing up to 5100 GWh per year; https://www.sitecproject.com), small distributed energy sources are still being developed throughout the province. As such, BC can serve as an example for other jurisdictions aiming at meeting their energy demand via small, distributed renewable sources. Simultaneously large shale gas deposits are being rapidly developed in BC (half of Canada’s shale gas reserves, estimated at ~1200 trillion cubic feet^33^). However, neither form of new energy development is currently regulated by land use planning that accounts for the potential for regional biodiversity impacts concomitant with meeting GHG emissions targets. This is an important gap, as British Columbia harbors some the last pristine ecosystems and large mammal assemblages in North America, free-flowing salmon rivers, while many ecosystems and iconic species are under threat from land conversion and other forms of human impacts, including energy development^34–36^.

In this study we quantified the potential trade-offs between development of three electricity-generation technologies [renewable: river diversion hydropower, hereafter *run-of-river hydro*, and on-shore wind, hereafter *wind farms*, and unconventional fossil: extraction and combustion of natural gas from shale], and biodiversity conservation objectives at regional (spatial conservation priorities) and global (GHG emissions) scales. The objectives of this study were to quantify the trade-offs among these energy technologies for biodiversity conservation using three common measures of potential biotic impact: (1) lifecycle GHG emissions for each technology (lifespan of electricity-producing technologies of 30–40 years), (2) cost per megawatt hour ($/MWh) of potential electricity development, and (3) the degree of spatial overlap between infrastructure associated with the future development of each energy technology and regional priorities for species conservation. Despite its widespread use in reserve design, conservation management, and urban planning^37^, spatial conservation planning has not yet been used to assess biodiversity trade-offs among different renewable and unconventional fossil fuel technologies, and this work has the potential to streamline decision-making on electricity portfolios in the energy planning process.

## Materials and Methods

### Analysis workflow

This study was focused on the Canadian province of British Columbia, which covers 1 million km^2^, and has strong latitudinal, altitudinal, and longitudinal environmental gradients (from temperate rainforest to boreal forest, semi-desert, and dry grasslands). BC encompasses the most extensive undisturbed temperate rainforests, and some of the last free flowing rivers and intact vertebrate food webs in temperate North America. At the same time, much of the province has been under intense resource extraction pressure (timber, mining, natural gas) for nearly a century, and such extraction continues to contribute substantially to a growing domestic economy as well as foreign trade.

We used existing Life Cycle Assessment (LCA) studies to predict future GHG emissions for wind farms and run-of-river hydro, and combined combustion and upstream emission estimates for future projected natural gas development (e.g., shale gas extraction, processing, and transport). Second, we extracted the costs of electricity production ($/MWh) from both renewable sources, and from natural gas combustion from an existing dataset of all potential electricity sources, used in strategic energy planning in British Columbia^38^; we limited the dataset to potential development sites with electricity production costs of < $150/MWh to reflect current electricity markets^39^. Lastly, we estimated the spatial overlap of each energy technology with areas of high conservation priority in BC derived using systematic conservation planning principles^40^. We identified areas of high conservation value drawing on 385 vertebrate species distributions (341 small-bodied terrestrial species, 7 large-bodied carnivores and ungulates, and 37 fish species) and existing anthropogenic disturbance (i.e., forestry and linear infrastructure) under five prioritization scenarios: each of the three sets of species and anthropogenic disturbance separately, and a scenario combining all four datasets.

### Energy datasets

**Figure 1:**
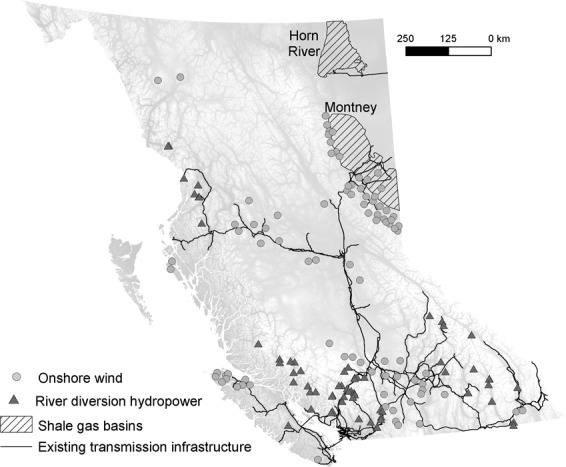
Spatial distribution of potential run-of-river hydro and wind farm facilities (projects < $150/MWh), and shale gas basins in British Columbia. Run-of-river hydro and wind farm data from BC Hydro Resource Options Report (2013); shale basins data from DataBC (https://apps.gov.bc.ca/pub/dwds/home.so). Maps produced using ArcGIS 10.5 (ESRI, Redlands CA, USA).

To evaluate how potential energy development overlaps with areas of high conservation priority in BC we used a spatially-explicit dataset produced by the BC Provincial power utility BC Hydro (^38^; https://catalogue.data.gov.bc.ca/dataset/bc-hydro-resource-options-mapping-2013) that identified locations of potential electricity production (wind farms and run-of-river hydro) infrastructure^38^. Specifically, BC Hydro used topographic and climatic factors to identify the approximate sitting of individual renewable energy projects, as well as the spatial location of potential infrastructure associated with each project, such as access roads connecting each project to the nearest road network, and powerlines connecting each project to the provincial power grid^38^. While other energy technologies have been identified, wind farms and run-of-river hydro represent more than half of the potential renewable electricity generation in BC^38^, and nearly 8,000 potential development locations. We set a maximum development cost of $150/MWh to limit our analysis to a subset of economically viable projects, resulting in 66 run-of-river hydro locations and 87 wind farm locations (Fig. 1). We chose this threshold to be greater than the highest price paid for renewable energy in BC (up to $124/MWh in 2017) to allow for increases in the cost of electricity in the near future, but also to exclude sites that are cost-prohibitive in the near term. This threshold also reflects renewable electricity costs worldwide, which have been decreasing over the past two decades, and are forecast to decrease in the near term^39^. Energy cost estimates for wind and run-of-river locations are amortized for the lifetime of the projects (~30 years), include all infrastructure development (i.e., roads, power generation facilities, and powerlines), and are expressed in 2013 dollars^38^.

For each renewable energy technology, we considered the aggregate spatial footprint of run-of-river hydro and wind farm potential development locations along with their associated linear infrastructure. For wind farms, we built buffers around each point location of individual wind farms equal in area to the estimated footprint of that particular project (9.0–96.0 ha per facility). For run-of-river hydro, we built buffers around the project locations equal to the estimated footprint of the generating infrastructure (i.e., powerhouse, dam and headpond; 2.3 to 12.9 ha per facility). In addition to these project footprints, we used the estimated spatial location of powerlines, access roads and, in the case of run-of-river, the water pipelines (referred to as penstocks) connecting the dam to the powerhouse^38^, to evaluate the overlap of renewable energy development with conservation priorities (see section *Evaluating overlap between energy development and conservation priorities*).

For natural gas-generated electricity, we considered the footprint of natural gas extraction from shale, transport pipelines, and four proposed gas-fired power plants (with a range of the range of electricity costs dependent on the technology used, and size of the plant). However, these natural gas power plants have a small spatial footprint relative to the footprint associated with shale gas extraction. For natural gas extraction from shale, we considered the two major gas basins under exploitation in BC covering ~3.9 million ha (Fig. 1) (Horn River and Montney), as well as approved, but not yet developed, rights-of-way for gas extraction, refinement, and transportation infrastructure (roads, pipelines; www.data.gov.bc.ca/).

### Life cycle assessment-based greenhouse gas emissions

We used GHG emission estimates from Life Cycle Assessment (LCA) expressed as grams of CO_2_ equivalent per kWh of energy produced for wind farms^41^, and run-of-river hydro^42,43^. LCA is a framework for evaluating environmental impacts of a project associated with all stages of its life, from extraction of raw materials, to manufacture, maintenance and disposal/recycling. For natural gas-generated electricity, we estimated GHG emissions by summing (1) estimates of the emissions generated from direct combustion^38^ with (2) estimates of upstream emissions (extraction, refinement, and transport of shale gas) produced using GHGenius 4.03 (www.ghgenius.ca). GHGenius is a peer-reviewed tool for estimating greenhouse gas emissions from a variety of fuels and industry sectors developed for Natural Resources Canada^44^. GHGenius is based on an existing Lifecycle Emissions Model^45^, and focuses on the lifecycle assessment of current and future fuels in transportation and electricity generation.

### Biodiversity metrics

#### Small-bodied terrestrial vertebrates

We gathered occurrence locations for terrestrial vertebrate species from two open-access online databases: Global Biodiversity Information Facility (*data.gbif.org*) and Nature Counts (*birdscanada.org/birdmon*). We excluded species with fewer than 100 occurrence points within BC, which resulted in 341 native vertebrate species (15 amphibians and reptiles, 25 mammals, and 301 birds; Supporting Information S1) modeled using ensemble models^46^ implemented in program R^47^ via the biomod2 package^48^ (see Supporting Information S2).

#### Freshwater fish species

We used fish habitat suitability models for 37 species, including five native anadromous salmonids, *Oncorhynchus spp*. developed by the BC Ministry of Environment in support of the provincial Fisheries Sensitive Watersheds initiative (http://www.env.gov.bc.ca/wld/frpa/fsw/). These habitat models relate species occurrences in BC streams and rivers to stream and watershed physical attributes and ecological processes at the scale of subwatershed units (mean area = 4,920 ± 78 ha^49^).

#### Large-bodied carnivores and ungulates

We selected species that can be sensitive to broad-scale land-use development and that have well-documented range estimates. These constraints allowed us to consider to seven large mammals; bighorn sheep (*Ovis canadensis*), caribou (*Rangifer tarandus caribou*), elk (*Cervus canadensis*), fisher (*Pekania pennanti*), mountain goat (*Oreamnos americanus*), grizzly bear (*Ursus arctos*), and wolves (*Canis lupus*). Building on continental range estimates in previous work^36^, we refined each range map to British Columbia based on expert consultation within relevant government research bodies and the most recent status reports from the BC Ministry of Forests, Lands, and Natural Resources (bighorn sheep and elk^50,51^; fisher^52^; wolf^53^). These range maps were initially created as raster with 250 × 250 m cell size^34^, then rescaled to a 400 × 400 m resolution for use in the prioritization scenarios.

#### Existing landscape disturbance

Accounting for past landscape disturbance is critical for correctly identifying conservation priorities^54^. The province of BC has a relatively short (<100 years), but intensive history of landscape disturbance through resource extraction activities, including logging, mining, and oil and gas extraction. As a result, certain areas of the province have dense networks of infrastructure (roads, powerlines, pipelines, seismic lines), while other, less accessible and remote regions, are still relatively undisturbed by human activities. To account for legacies of past human disturbance, we used two landscape disturbance indicators summarized at the sub-watershed level (n = 19,469, mean area = 4,920 ± 78 ha), density of linear fragmentation (km/km^2^), and recent forest loss (1990–2012) from logging, wild fires, and pests (i.e., pine beetle). We used the Digital Road Atlas of British Columbia (http://geobc.gov.bc.ca/base-mapping/atlas/dra/), along with existing transmission and distribution lines^38^ to create a dataset of existing linear infrastructure in the province. We created a 100-m resolution layer of forest cover change between 1990 and 2012 by combining two spatial data sources: the 2013 Vegetation Resources Inventory (BC Ministry of Lands, Forests and Natural Resources Operations), a vector dataset of forest cover and logging activities in BC, and the Global Forest Change project^55^, a 30-m resolution global dataset of forest cover gains and loses derived from Landsat satellite imagery.

### Overlap between energy development and conservation priorities

#### Identifying spatial conservation priorities

We used the systematic conservation planning methods and software Zonation v. 4^56^ to identify top spatial conservation priorities in British Columbia under five scenarios that considered: (1) terrestrial vertebrate species (341 birds, amphibians, reptiles, and mammals [excluding large mammals]), (2) seven large mammal species, (3) 37 fish species, including the five species of anadromous salmon, (4) existing landscape disturbance from linear fragmentation (such as roads, pipelines, seismic lines, railroads) and recent forest loss (1990–2012, from logging, wildfires, and pests), and (5) a combination of the four previous species and landscape disturbance scenarios. Under the landscape disturbance scenario (scenario 4), conservation priorities were identified as areas that are intact (undisturbed habitat), as a proxy for intact species assemblages and food webs. For individual taxonomic group scenarios (1–4), each species was weighed equally within its own prioritization scenario. For the combined scenario, each of the four taxonomic and disturbance datasets were weighed equally; for example, each of the 341 terrestrial vertebrate species (dataset 1) were given a weight of 0.00294 (thus summing to 1), while each of the two disturbance layers in dataset 4 was given a weight of 0.5. This weighting system led to a balanced representation of the intact landscapes and the three different sets of species with different habitat and space requirements. When all four sets of data were combined, Zonation produced conservation prioritization outputs that maximized both biodiversity value (e.g., for each taxonomic dataset), and areas with the most undisturbed habitat.

Zonation produces a complementarity-based and balanced ranking of conservation priorities across the entire landscape^57^. Zonation calculates a rank order value of each cell in the landscape based on conservation priority (ranging from 0 = *low conservation priority* to 1 = *high conservation priority*). As a general rule, cells with rank values > 0.8 are considered to be of high conservation priority. Zonation is able to combine probabilistic species distributions data, large raster datasets (>10^8^ grid elements), and can balance the distribution of species or communities with connectivity, costs, and needs of alternative land uses in the same prioritization^58^. For the purpose of this study, we used the additive-benefit function of Zonation with an exponent *z* = 0.25. Under this function, conservation value is additive across biodiversity features, and individual feature representation is converted via a benefit function that is in shape the same as the canonical species-area relationship (concave power function^59^). We also took into account existing protected areas, which cover ~10% of BC, using a hierarchical mask^60^. A hierarchical mask defines a strict sequence of cell removal, starting with the lowest values. In our case, the hierarchical mask was given a value of 1 for all protected areas, and 0 for the rest of the province. Thus, protected areas were given the highest conservation rank (top 10% of the province). The reasons for applying the hierarchical mask are twofold: (1) BC Hydro’s renewable energy planning process, as well as shale development, avoid existing protected areas, and (2) we aimed to focus our analyses on areas currently at risk from future energy development decisions. We excluded from the analyses all areas in the province covered by exposed rock, glaciers, barren lands, lakes, and urban areas. All the species, communities, ecosystems, and disturbance data were processed at a resolution of 400 × 400 m, with a resulting landscape of 5,131,698 cells with data.

#### Evaluating overlap between energy development and conservation priorities

Comparing the potential impacts of renewable and unconventional energy development poses several challenges. Foremost, our dataset on potential renewable energy development locations allowed us to calculate the length of new roads and powerlines, as well as the areal footprint of wind farm and run-of-river facilities, but there is no parallel dataset for future shale development. To evaluate the overlap between energy development and conservation priorities, we extracted the Zonation rank values for each cell intersected by the infrastructure of the 66 run-of-river hydro projects (n = 6,623 cells, of which 362 cells were buffer-based footprints, and the rest represented overlap with run-of-river linear infrastructure) and 87 wind farms (n = 13,383 cells, of which 1,240 cells were buffer-based footprints, and the rest represented overlap with wind farm linear infrastructure).

For shale development, we combined the locations of the two shale gas basins (Fig. 1) with the spatial footprint of approved, but not yet developed, rights-of-way for gas extraction, refinement, and transportation infrastructure outside the shale basins. Unconventional oil and gas extraction results in a network of well pads, access roads, pipelines, powerlines and seismic lines that tends to cover broad spatial areas^61^ compared to highly localized wind farms and run-of-river hydro and their estimated linear infrastructure. In addition, within the two BC shale gas basins, the exact footprint and location of future shale development is uncertain, and extraction activities are by nature, distributed across many individual wells. In the absence of exact locations of shale development infrastructure within the two shale basins, we randomly selected 10,000 points at which to extract ranks for each prioritization scenario and characterized the variation in Zonation ranks associated with current and future shale development. While this approach is different from the renewable energy footprint, the outcome (i.e., range of conservation ranks overlapped by energy infrastructure) is comparable, as we did not attempt to compare the three energy technologies in terms of their absolute footprint.

We quantified the overlap between potential energy development and conservation priorities produced from the Zonation outputs using probability density plots for the frequency of cells intersected by potential development across the range of Zonation ranks. A skewed distribution of Zonation ranks towards the highly-ranked cells (i.e., >0.7) is indicative of potential conflicts between conservation and energy development. For each energy technology, we calculated the proportion of cells corresponding to wind (13,383 cells), small hydro (6,623 cells) and shale development (10,000 cells) with conservation ranks > 0.7 under each prioritization scenario. Using density plots across the range of Zonation ranks instead of absolute measures of overlap (e.g., km^2^ impacted) allowed us to examine the relative overlap of potential shale development with conservation priorities without having to make assumptions about the future locations or density of shale infrastructure, and allowed a direct comparison with run-of-river hydro and wind farms.

We calculated the amount of renewable electricity that could be developed without impacting high conservation priority areas under each scenario (Zonation rank > 0.7, instead of >0.8, because protected areas accounted for the top 10% [0.9–1.0] of Zonation scores after applying the hierarchical mask^60^). While the overlap analysis evaluated trade-offs between the three energy sectors, this analysis provides complementary information, as it focuses on individual renewable energy projects by quantifying the potential energy gain from projects located in low conservation value areas. For each individual renewable energy project, we calculated the average and the range of Zonation ranks of cells intersected by its infrastructure (see above for description of infrastructure considered for potential renewable energy projects). We then ranked the 87 potential wind farms and 66 run-of-river hydro projects separately by their average Zonation rank value, and plotted the cumulative gain in annual electricity potential relative to the average and range of Zonation values.

## Results

**Figure 2:**
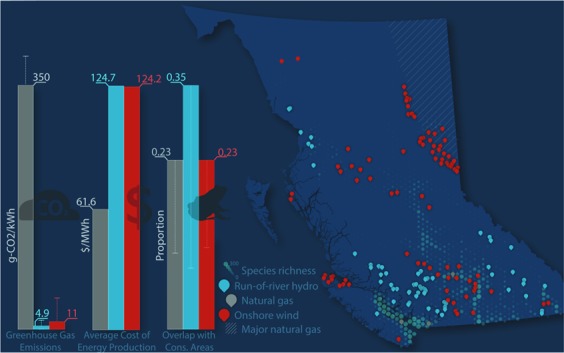
Comparison of electricity cost, GHG emissions, and overlap with high-conservation-value areas across potential run-of-river hydro, wind farms, and shale gas development. The cost of enegy production is the weighted mean cost of development ($CAD/MWh) across all potential locations. The overlap with high-conservation-value areas is the proportion of cells with Zonation rank > 0.7 that is overlapped by the infrastructure and development footprint for each energy source. GHG emissions are the per unit energy estimates of CO_2_ from lifecycle analyses for run-of-river hydro and wind farms, and the sum of combustion and upstream emissions (e.g. extraction, processing, and transport) for shale gas development. Figure produced in Adobe Creative Suite 6 (Adobe Systams Inc, San Jose CA, USA).

We identified strong trade-offs between the potential regional and global biodiversity impacts of future electricity production in BC. Specifically, we found that when we predicted areas of high biodiversity value across the vertebrate species groups and undisturbed landscape, potential wind farm locations and shale gas extraction locations had similarly low-to-medium overlap (0.06–0.36 and 0.07–0.43% of cells with Zonation rank > 0.7 for wind farms and shale development, respectively, with the highest values associated with the large mammal scenario; Table 1; Fig. 2). In contrast, potential run-of-river hydro development locations and infrastructure overlapped the most with high conservation priorities (up to 0.7 under the small-bodied vertebrates scenario; Table 1), had an average energy cost (128.4 $/MWh), competitive with the current renewable electricity market, and had the lowest GHG emissions per unit electricity. As expected, the extraction and combustion of natural gas from shale deposits had the highest potential for future GHG emissions (310–367 g CO_2_-eq/kWh), up to 1000 times higher than what we estimated for run-of-river hydro or wind energy (0.3–13 and 3–45 g-CO_2_/kWh respectively), but could be produced for the least cost (mean = 90.6 $/MWh) (Fig. 2). Upstream emissions (from extraction, transport, and processing) constituted 8–15% of the GHG emissions for shale gas, with the remainder resulting from natural gas combustion. Considering these trade-offs between economics of energy development and regional and global conservation priorities, we found that wind energy has the highest potential for energy production at a cost competitive with current renewable electricity market prices (average = 126.5 $/MWh), while minimizing GHG emissions (3–45 g-CO_2_/kWh), and avoiding overlap with most high priority areas for biodiversity protection in BC (0.06–0.36; Table 1).

**Table 1:** Spatial overlap between three energy technologies, and spatial conservation priorities in British Columbia: proportion of cells (400-m) for each energy technology (Run-of-river = 6,623 cells; Wind farm = 13,383 cells; shale gas = 10,000 cells) with Zonation scores of > 0.7 across five prioritization scenarios.

		Proportion energy technology footprint overlap with high conservation value areas (Zonation cells rank > 0.7)				
		Prioritization scenario				
		Landscape disturbance	Fish	Large mammals	Small-bodied vertebrates	All datasets
Energy technology	Run-of-River Hydro	0.10	0.34	0.05	0.70	0.56
	Wind Farms	0.06	0.22	0.36	0.28	0.24
	Shale Gas	0.07	0.29	0.43	0.11	0.23

### Spatial conservation priorities in BC

**Figure 3:**
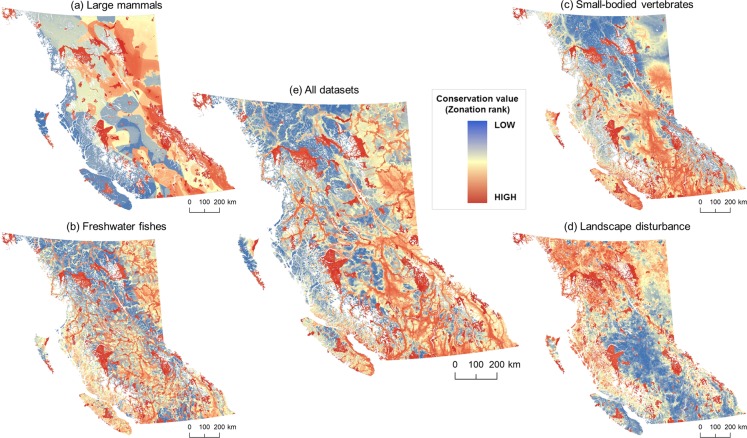
Spatial tradeoffs between conservation priorities under five prioritization scenarios (**a**) seven large, wide-ranging mammals, (**b**) 37 freshwater fishes, (**c**) 341 small-bodied vertebrates, (**d**) intact landscapes, and (**e**) all datasets combined and equally weighed. Protected areas are always given the highest conservation priority across all scenarios. Prioritization scenarios were implemented in software Zonation v4; maps produced using ArcGIS 10.5 (ESRI, Redlands CA, USA).

**Figure 4:**
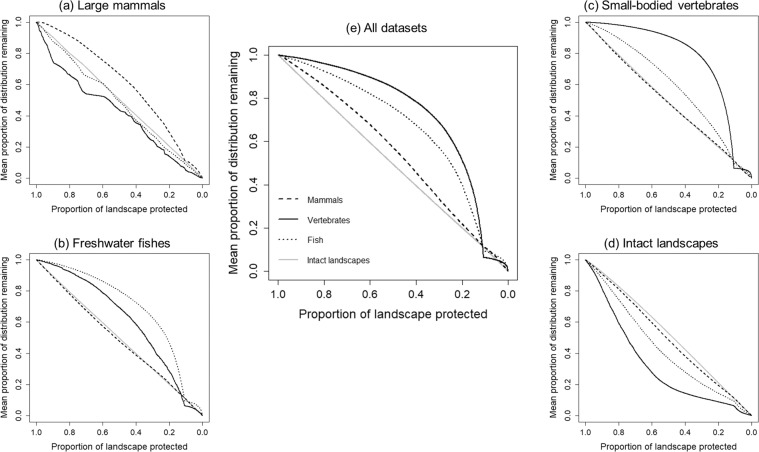
Performance curves quantifying the average proportion of the original distributions represented at each fraction of the landscape retained for conservation. Under the five scenarios, we evaluated (**a**) whether protecting seven large mammals performs well for protecting other terrestrial and aquatic vertebrate species and intact landscapes, (**b**) whether protecting 37 freshwater fishes performed well for protecting large and small bodied-vertebrates and intact landscapes, (**c**) whether protecting 341 small-bodied vertebrates performed well for protecting large mammals, freshwater fishes and intact landscapes, (**d**) whether intact landscapes (with less forest loss and linear disturbance) perform well for protecting terrestrial and aquatic vertebrates, and (**e**) how well individual species groups and intact landscapes are protected when considered simultaneously. A higher proportion of landscape protected at a higher level of distribution retained denotes greater performance at representing a given species group or intact landscapes. For example, protecting least disturbed areas (**d**) performed poorly for all species groups.

When the four datasets (large mammals, small-bodied vertebrates, freshwater fishes, and intact landscapes) were considered individually, we found that the spatial conservation priorities identified were geographically distinct (Fig. 3a,b,c,d). We found that the fifth scenario, which combined all vertebrate species and undisturbed landscapes, provided a balance between the other four scenarios (Fig. 3e). After discounting existing protected areas, which had the highest Zonation scores (>0.9), the top spatial conservation priority areas for the large mammal-only scenario (Zonation rank 0.7–0.9; i.e., top 20% conservation priority area not currently protected from development) were concentrated in the Southern Rockies and North-Central BC (Fig. 3a); priorities for small-bodied vertebrates were concentrated in Central and Southern BC, Southern Vancouver Island, and valley bottoms in the Rockies, and Coast Mountains (Fig. 3). In contrast, priorities for conserving undisturbed landscapes without considering vertebrate species distributions were concentrated in Northwestern BC and the Central Coast (Fig. 3d), and priorities for freshwater fishes were more dispersed across geographic regions (Fig. 3b). Overall, prioritizing any given dataset performed poorly for the other datasets, suggesting limited overlap in biogeography and low potential for surrogacy (i.e., protecting a particular taxa conferring protection to other species^62^; Fig. 4a,b,c,d). For example, prioritizing small-bodied vertebrates failed to provide a conservation benefit to large mammals or intact landscapes, and provided only a moderate benefit for freshwater fishes (Fig. 4c). Similarly, areas identified as important for large, wide-ranging carnivores and ungulates did not capture species rich areas for either small-bodied vertebrates or freshwater fishes (Fig. 4a). The prioritization scenario (5) that included all four datasets provided a balance in representing all biodiversity features included in the analysis (Fig. 4e), although it performed less well for large mammals and intact landscapes.

#### Overlap between energy development and conservation priorities

We found that the three energy technologies were geographically segregated from one another, with shale development (extraction) occurring in relatively low elevation and flat areas of Northeastern BC, run-of-river hydro on streams in the coastal mountains and southern Rocky Mountains, and wind farms at higher elevations in the northern Rockies and central-southern BC, as well as Vancouver Island (Fig. 1). According to BC Hydro estimates, the 66 potential run-of-river hydro projects included in our analyses would require 2,858 km of new powerlines and 174 km of new roads, and a physical footprint of the actual projects of 579.5 ha (range = 2.3–12.9 ha/project). The 87 wind farms in our dataset would need 5,460 km new powerlines and 451 km of new roads, and would cover 1984 ha (range = 9–96 ha/project). The two shale gas basins are much larger (~3,900,000 ha) relative to the spatial footprint of potential renewable energy (wind farms, run-of-river hydro) development. Currently, shale gas extraction occurs on ~2,700,000 ha in Northeastern BC, with a total gas production of ~59 billion m^3^ in 2018 (with an increasing trend from ~32 billion m^3^ in 2007; http://www2.gov.bc.ca/gov/content/industry/natural-gas-oil/statistics). Of this amount, approximately 3% (~1.6 billion m^3^) would be needed to power the four potential natural gas-fired plants under consideration in this study (for a total of ~5,500 GWh/year), assuming that 0.2862 m^3^ of natural gas is required to produce 1 kWh of electricity (US Energy Information Administration; www.iea.gov). This highlights that the footprint of natural gas electrification plants represents a relatively small contribution to the overall development footprint of shale gas (as most of the shale gas is exported, not used in electricity generation in BC), as well as the substantial uncertainty that exists in determining the specific areas that would be disturbed from extracting natural gas specifically for electricity production. Shale gas extraction activities in the two shale basins, Montney and Horn River, had moderate overlap with large mammals (proportion of cells with rank > 0.7 = 0.43; Fig. 2, Figure S1, Table 1), and little overlap with small-bodied vertebrates, fish, or landscape disturbance (proportion of cells with rank > 0.7 = 0.07–0.29; Fig. 2, Figure S1, Table 1).

**Figure 5:**
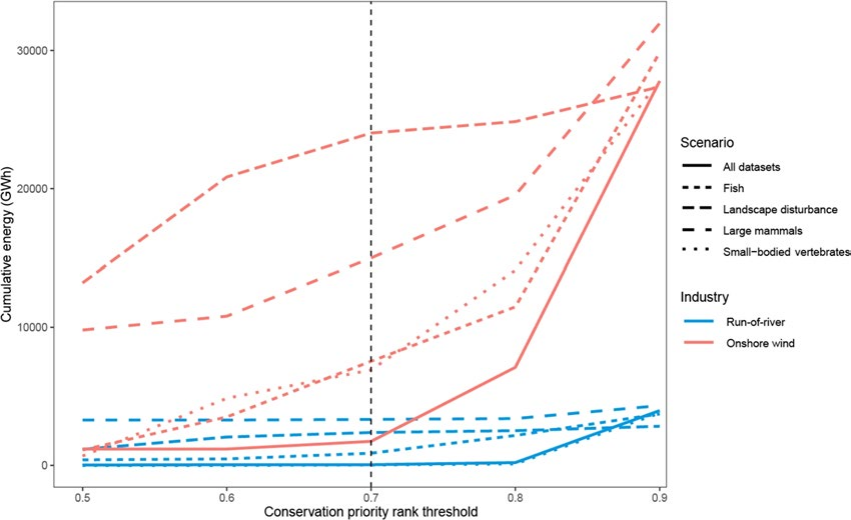
Cumulative energy gain from run-of-river hydro (blue) and wind farms (red) relative to the spatial overlap between energy infrastructure and conservation priorities under five prioritization scenarios. Projects falling between Zonation ranks 0.7 (dotted vertical line) and 0.9 have high overlap with spatial conservation priorities in each scenario. Wind farms with low overlap with conservation priorities (<0.7) have the potential produce significantly more electricity compared to run-of-river hydro projects.

The annual firm energy gained from developing all 66 run-of-river hydro projects would be 4,762 GWh/year. These potential locations and their infrastructure had high overlap with high value conservation areas for small-bodied vertebrates (proportion of cells with rank > 0.7 = 0.7; Figs. 2, S1; Table 1). In contrast, when prioritization was based on currently undisturbed areas or large mammals, there was less overlap between run-of-river hydro infrastructure and areas of high conservation value (0.1 and 0.05 proportion of cells with rank > 0.7, respectively). Run-of-river hydro projects that could be built outside high value conservation areas across the five prioritization scenarios could generate up to ~4,000 GWh/year of electricity (Fig. 5). In contrast, potential wind farm locations had less overlap with high value conservation areas compared to run-of-river hydro across all prioritization scenarios (proportion of cells with Zonation rank > 0.7 = 0.06–0.36; Figure S1, Table 1). The 87 wind farms that could be developed for < $150/MWh have the potential to generate a total of 35,428 GWh/year of new electricity. Of these, ~6,000–24,000 GWh/year can be developed in low conservation value areas, depending on the species and disturbance-specific conservation prioritization scenario, with the highest output and lowest overlap for the undisturbed landscapes scenario (Fig. 5). However, both run-of-river and wind farms have the potential generate very little energy in low conservation value areas when all datasets are considered simultaneously (combined prioritization scenario; Figs. 3e, 5). Because the shale gas production was considered cumulatively across the two shale gas basins (rather than a well-by-well, or lease basis), it was not possible to make a similar comparison to estimate the amount of gas (and associated energy) produced in low conservation value areas.

## Discussions

Greenhouse gas emissions (carbon dioxide, methane, nitrous oxide, etc.) from human activities are the main driver of current climate warming, with energy and transportation sectors accounting for more than half of global emissions^63,64^. As expected, our analysis confirms that GHG emissions were much greater for shale gas extraction and combustion compared to the two renewable energy technologies, but the differences in overlap between shale gas, run-of-river hydro, and wind farms with regional conservation priorities (Figure S1; Table 1) highlights a trade-off for biodiversity conservation between global and regional scales. In our analyses, there were substantial spatial disparities between the top conservation priorities when each species group and intact landscapes were considered individually (Fig. 3a,b,c,d). While recent studies have evaluated geographic differences in sitting of new energy projects^13,65^, studies that focus either on GHG emissions or biodiversity impacts may miss important trade-offs between global and regional conservation targets and priorities. Such underlying trade-offs are important, yet rarely quantified or acknowledged, when thinking about avoiding or minimizing overlap between energy development and regional conservation priorities.

In British Columbia, shale gas extraction is concentrated in two basins in the northeastern part of the province, a region of low vertebrate species richness, but with low levels of existing disturbance and moderate conservation value for freshwater fishes and large mammals (Figs. 1, 3). Many species with large space requirements (e.g., grizzly bear (*Ursus arctos*), caribou (*Rangifer tarandus*)), that also have high value to local communities, could be impacted by habitat fragmentation associated with shale development^14,15^, and additional shale development has high potential for cumulative effects at the population level^66^. Globally, shale gas formations are estimated to cover >20 million km^2^ ^33^, and overlap with areas of high aquatic and terrestrial biodiversity^14^. At the same time, the GHG emissions from shale gas development are similar to those of conventional natural gas, though when used for electricity generation, GHG emissions are half of those from coal-fired plants^5^. Thus, without global emission-reduction measures (e.g., carbon capture and storage, alternative transportation fuels), electricity production from shale gas will increase global GHG emissions and contribute to long term temperature rise^5^, impacting biodiversity worldwide^67,68^.

Both run-of-river hydro and wind energy technologies had much lower lifetime GHG emissions than shale gas extraction and combustion, but they differed in their potential spatial overlap with areas of high conservation priority across species groups and intact landscape metrics (Fig. 2, Figure S1). Run-of-river hydro development has the highest development potential on montane streams (e.g., Coast Mountains, Southern BC Rockies; Fig. 1). We found that potential powerlines and roads associated with run-of-river hydro facilities in these regions are often concentrated in valley bottoms^38^, which have high species richness and house unique terrestrial vertebrate communities, thus increasing overlap with high priority conservation areas for small bodied vertebrates. The Southern Rockies also have high conservation value for large carnivores and ungulates, further highlighting potential conflicts with species sensitive to terrestrial habitat fragmentation and disturbance. In contrast to run-of-river hydro, potential wind farms are concentrated in coastal areas (northern Vancouver Island) and at higher elevation in the southern Interior of BC and the Central Rockies (Fig. 1), although their associated powerlines also tend to converge in valley bottoms. Regardless of the prioritization scenario, wind farm locations had relatively low overlap with high conservation value areas (Table 1), suggesting that potential wind energy development presents less conflict with terrestrial and aquatic biodiversity than the other energy technologies considered here. Overall, selecting renewable energy project locations is more flexible compared to shale development. While we focused on a particular set of renewable energy projects in this study, additional wind and small hydropower sites could be identified to alleviate regional conservation concerns (likely with added economic costs); shale development does not afford this flexibility, as it has to overlap with the geography of shale deposits.

The combined prioritization scenario (5) suggests that that energy development planning in British Columbia could consider both species-rich areas for terrestrial vertebrates, freshwater fishes, and intact landscapes simultaneously, but individual prioritizations are useful for evaluating potential overlap with areas of high conservation values for species groups that have high importance for local communities (e.g., freshwater fishes, Fig. 4b), and intact landscapes (Fig. 4d).

### The need for cross-scale evaluation of biodiversity impacts from energy development

In spite of the worldwide boom in renewable energy development, as well as shale gas extraction, the trade-offs between protecting biodiversity at local or regional scales versus measures aimed at protecting biodiversity globally remain largely unquantified. The exponential increase in renewable energy development worldwide (including solar and geothermal, not considered in this study^4^), has recently been accompanied by evaluations of impacts on fish and wildlife. Studies of impacts of wind energy on biodiversity have focused on bird and bat mortalities at wind turbines^69–71^, leading to changes in mitigation measures required to reduce such impacts, such as decreasing the rotation speed, placing facilities outside migration routes^72^, or developing best management practices for sitting wind farms that minimize impacts to wildlife^73,74^. Fewer studies have investigated the biodiversity impacts of run-of-river hydro^75,76^. Similarly, the potential for impacts from shale development on fish and wildlife has only been studied superficially, and surprisingly large gaps exist in our knowledge of impacts to biodiversity, particularly around ground and surface water contamination from fracturing fluids, terrestrial fragmentation from infrastructure, and cumulative impacts^14^. Our study provides a platform for future studies to incorporate uncertainty in renewable and unconventional energy development locations, and their potential biodiversity impacts, while simultaneously considering their contribution to GHG emissions, as well as economic competitiveness. A natural extension of this work would be to identify portfolios of renewable and shale development projects that minimize trade-offs between energy production and local impacts on species of conservation concern, as well as species and landscapes of high social and cultural value for settler and First Nation communities. The three metrics considered here (GHG, cost and species conservation) provide contrasting perspectives on energy development, but the range of potential metrics for investigating trade-offs between renewable and fossil fuels is considerably greater. In particular, the current study does not acknowledge social aspects, such as attitudes towards different types of energy development, or cultural, recreational, and spiritual values of settler and First Nation communities. Tackling such additional complexity and identifying favored energy solutions requires thoughtful engagement with diverse groups in a participatory decision-making framework.

At a global scale, lowering the GHG emissions of the energy sector is a critical step towards stabilizing global temperatures^63^, and requires a fundamental transformation of the global energy production system. While some progress has been made at reducing GHG emissions among developed countries since the 1990’s (e.g., −20% in the European Union, no change in the United States, +19% in Canada), the energy sector remains the greatest source GHG emissions globally (~30%). Along with increased efficiency and lower consumption rates, the Fifth Assessment Report of IPCC recommends that the efforts to reduce the GHG emissions from the energy sector focus on transitioning from coal-generated electricity to renewable electricity, as well as natural gas-powered plants (providing that upstream emissions from natural gas extraction become minimal)^63^. Concomitantly, some jurisdictions and energy sectors have been proactive in incorporating potential biodiversity impacts in distributed renewable energy planning (e.g., wind energy and solar energy in the U.S., onshore and offshore wind in the EU). However, for jurisdictions where energy planning relies largely on economic-only metrics, challenges remain to environmental assessment and planning for meeting energy demands from many small renewable energy facilities.

Development projects are generally considered individually in the environmental impact assessment process^77^, an approach that does not account for the potential cumulative impacts of multiple facilities across spatial scales or multiple energy sources^78^. In contrast, strategic-level assessment approaches evaluating the potential for cumulative impacts from many energy projects relative to pre-existing economic activities and future development^24^, must incorporate conservation and biodiversity as quantified objectives along with lifecycle GHG emissions and cost of energy. Our study complements existing strategic planning approaches to energy development and cumulative impact assessment (e.g.^25–27,79^), and provides a perspective that cuts across energy sectors and build from well-established systematic conservation planning principles. Evaluating the potential for biodiversity impacts from energy portfolios across multiple scales, species groups, and jurisdictions complements local and regional environmental assessments for individual energy projects, and can act as an important filter to inform strategic decisions for land allocation and energy policies.

## Supplementary information

Supplementary information.
