# Supplementary material for: Quantifying biodiversity trade-offs in the face of widespread renewable and unconventional energy development
Source: Sci Rep. 2020 May 5;10:7603. doi: 10.1038/s41598-020-64501-7 (PMC7200705; doi:10.1038/s41598-020-64501-7)
Supplement: Supplementary file 1 — Supplementary information. [file 41598_2020_64501_MOESM1_ESM.docx]

**SUPPORTING INFORMATION**

**Quantifying biodiversity trade-offs in the face of widespread renewable and unconventional energy development**

Viorel D. Popescu^1,2,3^, Robin G. Munshaw^2^, Nancy Shackelford ^4^, Federico Montesino-Pouzols^5^, Evgenia Dubman^2^, Pascale Gibeau^2^, Matt Horne^6^, Atte Moilanen^7,8^, Wendy J. Palen^2^

^1^ Department of Biological Sciences and Sustainability Studies Theme, Ohio University, 107 Irvine Hall, Athens OH, 45701, USA

^2^ Earth to Ocean Research Group, Department of Biological Sciences, Simon Fraser University, 8888 University Dr., Burnaby, BC V5A 1S6, Canada

^3^ Centre for Environmental Research (CCMESI), University of Bucharest, 1 N. Balcescu Blvd, Bucharest, Romania

^4.^ Ecology and Evolutionary Biology, University of Colorado Boulder, 4100 Discovery Dr, Boulder, Colorado, 80303

^5^ European Southern Observatory, Science Operation Software Department, Garching bei München, Germany

^6^ City of Vancouver, Climate Policy, British Columbia, Canada

^7^ Department of Geography and Geosciences, P.O. Box 64, FI-00014 University of Helsinki, Finland

^8^ Finnish Natural History Museum, P.O. Box 17, FI-00014 University of Helsinki, Finland

**Figure S1.** Spatial overlap between conservation priorities and three renewable and unconventional energy resources in British Columbia. Plots show the distribution of potential energy development cells (run-of-river hydro = blue, wind farms = red, shale gas = grey) as a function of their conservation rank in BC for three prioritization scenarios; existing disturbance (top), vertebrate species richness (middle), disturbance and species (bottom). High y-axis values (density) for an energy technology represent high proportional overlap with the respective range of conservation priorities, relative to the spatial extent of that industry. X-axis is the Zonation ranking of cells (0 – 1); dashed vertical line represents the 0.7 Zonation rank threshold.


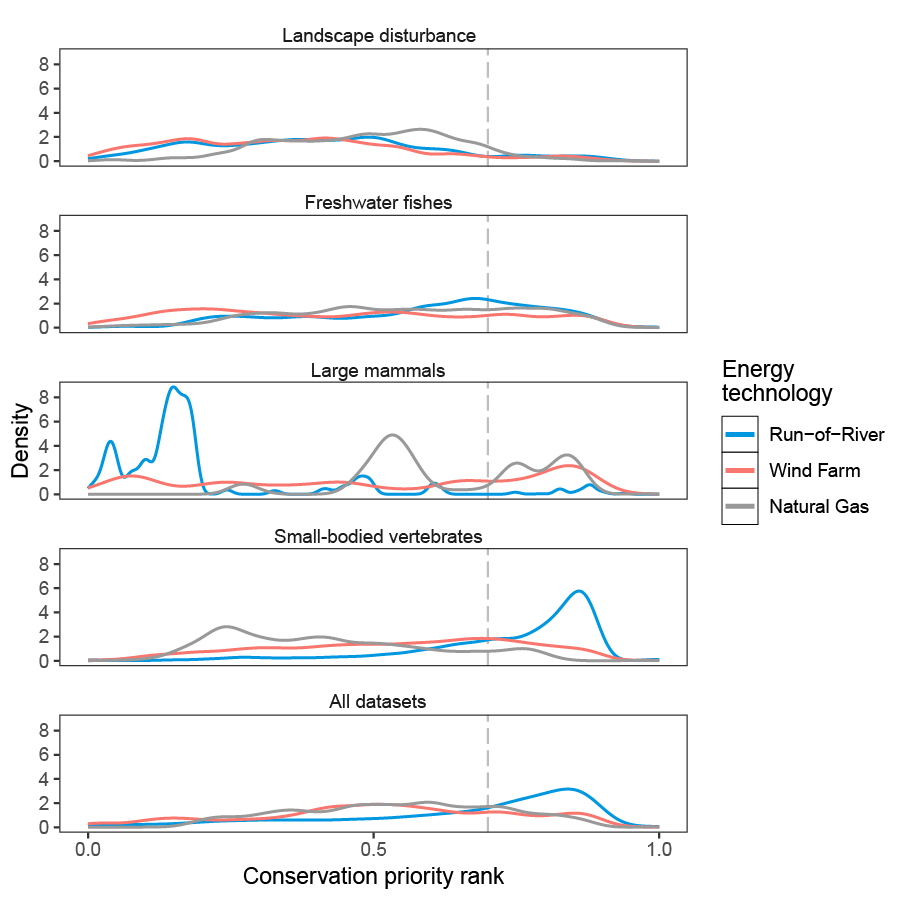


**Supporting Information S1.**

List of species included in the species prioritization (small-bodied terrestrial vertebrates, large mammals, and freshwater fishes) in British Columbia, Canada, and ensemble species distribution modeling performance summaries for small-bodied terrestrial vertebrate species.

Habitat classifications were determined semi-subjectively by considering habitat association summaries for each species. Aquatic species and large mammal distributions were acquired from an external source using different methods, so no modelling summaries were available. TSS = True Skill Statistic, Anad. Salmon = Anadromous salmonid. BC Provincial Conservation Status ranks: 1 = critically imperiled, 2 = imperiled, 3 = special concern, vulnerable to extirpation of extinction, 4 = apparently secure, 5 = demonstrably widespread, abundant, and secure, NA = not applicable, NR = unranked.

|  |  |  |  |  | **SDM performance summary** | | | |
| --- | --- | --- | --- | --- | --- | --- | --- | --- |
| **Species Name** | **Common Name** | **Taxon** | **Habitat Classification** | **BC Provincial Cons. Status** | ***# locations*** | **Ensemble TSS** | **Ensemble Sensitivity** | **Ensemble Specificity** |
| *Oncohynchus gorbuscha* | Pink salmon | Anad. Salmon | Aquatic | 5 | - | - | - | - |
| *Oncorhynchus keta* | Chum salmon | Anad. Salmon | Aquatic | 5 | - | - | - | - |
| *Oncorhynchus kisutch* | Coho salmon | Anad. Salmon | Aquatic | 4 | - | - | - | - |
| *Oncorhynchus mykiss* | Steelhead | Anad. Salmon | Aquatic | 5 | - | - | - | - |
| *Oncorhynchus nerka* | Sockeye salmon | Anad. Salmon | Aquatic | 4 | - | - | - | - |
| *Oncorhynchus tshawytscha* | Chinook salmon | Anad. Salmon | Aquatic | 4 | - | - | - | - |
| *Accipiter cooperii* | Cooper's hawk | Bird | Forest generalist | 4 | 5309 | 0.93 | 0.97 | 0.96 |
| *Accipiter gentilis* | Northern goshawk | Bird | Forest generalist | 2 | 2195 | 0.79 | 0.98 | 0.81 |
| *Accipiter striatus* | Sharp-shinned hawk | Bird | Forest generalist | 5 | 3789 | 0.86 | 0.95 | 0.91 |
| *Actitis macularius* | Spotted sandpiper | Bird | Non-forest | 5 | 6577 | 0.84 | 0.95 | 0.89 |
| *Aechmophorus clarkii* | Clark's grebe | Bird | Non-forest | 1 | 1180 | 1.00 | 1.00 | 1.00 |
| *Aechmophorus occidentalis* | Western grebe | Bird | Non-forest | 1 | 10173 | 0.95 | 0.99 | 0.96 |
| *Aegolius acadicus* | Northern saw-whet owl | Bird | Forest generalist | 5 | 2457 | 0.83 | 0.94 | 0.89 |
| *Aegolius funereus* | Boreal owl | Bird | Forest generalist | 4 | 143 | 0.71 | 0.91 | 0.80 |
| *Aeronautes saxatalis* | White-throated swift | Bird | Non-forest | 4 | 875 | 0.97 | 0.99 | 0.98 |
| *Agelaius phoeniceus* | Red-winged blackbird | Bird | Non-forest | 5 | 31859 | 0.91 | 0.99 | 0.92 |
| *Aix sponsa* | Wood duck | Bird | Forest generalist | 4 | 7984 | 0.95 | 0.98 | 0.97 |
| *Ammodramus savannarum* | Grasshopper sparrow | Bird | Non-forest | 1 | 108 | 0.99 | 1.00 | 0.99 |
| *Anas acuta* | Northern pintail | Bird | Non-forest | 4 | 6361 | 0.93 | 0.95 | 0.98 |
| *Anas americana* | American widgeon | Bird | Non-forest | 5 | 15824 | 0.92 | 0.95 | 0.97 |
| *Anas clypeata* | Northern shoveler | Bird | Non-forest | 5 | 6754 | 0.93 | 0.98 | 0.95 |
| *Anas crecca* | Green-winged teal | Bird | Non-forest | 5 | 12098 | 0.92 | 0.99 | 0.93 |
| *Anas cyanoptera* | Cinnamon teal | Bird | Non-forest | 4 | 2941 | 0.93 | 0.96 | 0.97 |
| *Anas discors* | Blue-winged teal | Bird | Non-forest | 4 | 2706 | 0.90 | 0.96 | 0.94 |
| *Anas penelope* | Eurasian widgeon | Bird | Non-forest | NA | 1053 | 0.96 | 0.97 | 0.99 |
| *Anas platyrhynchos* | Mallard | Bird | Non-forest | 5 | 44361 | 0.92 | 0.99 | 0.93 |
| *Anas strepera* | Gadwall | Bird | Non-forest | 5 | 9492 | 0.95 | 0.99 | 0.96 |
| *Anser albifrons* | Greater white-fronted goose | Bird | Non-forest | 4 | 909 | 0.94 | 0.99 | 0.95 |
| *Anthus rubescens* | American pipit | Bird | Non-forest | 5 | 2545 | 0.84 | 0.93 | 0.91 |
| *Aquila chrysaetos* | Golden eagle | Bird | Forest generalist | 4 | 1374 | 0.85 | 0.95 | 0.90 |
| *Archilochus alexandri* | Black-chinned hummingbird | Bird | Forest generalist | 4 | 1684 | 0.96 | 0.99 | 0.97 |
| *Ardea herodias* | Great blue heron | Bird | Forest generalist | 3 | 46397 | 0.93 | 0.97 | 0.96 |
| *Arenaria melanocephala* | Black turnstone | Bird | Non-forest | 4 | 313 | 0.97 | 0.98 | 0.99 |
| *Asio flammeus* | Short-eared owl | Bird | Non-forest | 3 | 550 | 0.88 | 0.96 | 0.92 |
| *Asio otus* | Long-eared owl | Bird | Forest generalist | 4 | 291 | 0.96 | 0.99 | 0.97 |
| *Aythya affinis* | Lesser scaup | Bird | Non-forest | 4 | 6552 | 0.91 | 0.98 | 0.93 |
| *Aythya americana* | Redhead | Bird | Non-forest | 4 | 4322 | 0.95 | 0.98 | 0.97 |
| *Aythya collaris* | Ring-necked duck | Bird | Forest generalist | 5 | 9572 | 0.91 | 0.93 | 0.98 |
| *Aythya marila* | Greater scaup | Bird | Non-forest | 4 | 4207 | 0.94 | 0.96 | 0.98 |
| *Aythya valisineria* | Canvasback | Bird | Non-forest | 4 | 2360 | 0.94 | 0.98 | 0.96 |
| *Bombycilla cedrorum* | Cedar waxwing | Bird | Forest generalist | 5 | 14999 | 0.89 | 0.99 | 0.90 |
| *Bombycilla garrulus* | Bohemian waxwing | Bird | Forest generalist | 5 | 3579 | 0.89 | 0.96 | 0.93 |
| *Bonasa umbellus* | Ruffed grouse | Bird | Forest specialist | 4 | 3330 | 0.77 | 0.93 | 0.84 |
| *Botaurus lentiginosus* | American bittern | Bird | Non-forest | 3 | 1244 | 0.90 | 0.94 | 0.96 |
| *Brachyramphus marmoratus* | Marbled murrelet | Bird | Forest specialist | 3 | 224 | 0.96 | 1.00 | 0.96 |
| *Branta bernicla* | Brant | Bird | Non-forest | 3 | 139 | 0.97 | 0.98 | 0.99 |
| *Branta canadensis* | Canada goose | Bird | Non-forest | 5 | 33656 | 0.93 | 0.95 | 0.98 |
| *Branta hutchinsii* | Cackling goose | Bird | Non-forest | 3 | 1018 | 0.96 | 0.98 | 0.98 |
| *Bubo scandiacus* | Snowy owl | Bird | Non-forest | 3 | 149 | 0.91 | 0.95 | 0.96 |
| *Bubo virginianus* | Great-horned owl | Bird | Forest generalist | 5 | 3258 | 0.90 | 0.97 | 0.93 |
| *Bucephala albeola* | Bufflehead | Bird | Forest generalist | 5 | 15134 | 0.91 | 0.94 | 0.97 |
| *Bucephala clangula* | Common goldeneye | Bird | Forest generalist | 4 | 7935 | 0.90 | 0.96 | 0.94 |
| *Bucephala islandica* | Barrow's goldeneye | Bird | Forest generalist | 4 | 5725 | 0.86 | 0.94 | 0.92 |
| *Buteo jamaicensis* | Red-tailed hawk | Bird | Forest generalist | 5 | 18400 | 0.90 | 0.93 | 0.97 |
| *Buteo lagopus* | Rough-legged hawk | Bird | Non-forest | 2 | 1410 | 0.90 | 0.95 | 0.95 |
| *Buteo platypterus* | Broad-winged hawk | Bird | Forest generalist | 3 | 278 | 0.86 | 0.92 | 0.94 |
| *Buteo swainsoni* | Swainson's hawk | Bird | Forest generalist | 2 | 1306 | 0.92 | 0.98 | 0.94 |
| *Butorides virescens* | Green heron | Bird | Non-forest | 3 | 1643 | 0.99 | 1.00 | 0.99 |
| *Calcarius lapponicus* | Lapland longspur | Bird | Non-forest | NA | 468 | 0.87 | 0.93 | 0.94 |
| *Calidris alba* | Snaderling | Bird | Non-forest | 4 | 360 | 0.95 | 1.00 | 0.95 |
| *Calidris alpina* | Dunlin | Bird | Non-forest | 4 | 1082 | 0.97 | 0.98 | 0.99 |
| *Calidris bairdii* | Baird's sandpiper | Bird | Non-forest | UN | 864 | 0.93 | 0.95 | 0.98 |
| *Calidris himantopus* | Stilt sandpiper | Bird | Non-forest | NA | 251 | 0.98 | 1.00 | 0.98 |
| *Calidris mauri* | Western sandpiper | Bird | Non-forest | 4 | 1687 | 0.94 | 0.97 | 0.97 |
| *Calidris melanotos* | Pectoral sandpiper | Bird | Non-forest | 5 | 1453 | 0.93 | 0.96 | 0.97 |
| *Calidris minutilla* | Least sandpiper | Bird | Non-forest | 4 | 2156 | 0.92 | 0.97 | 0.95 |
| *Calidris pusilla* | Semipalmated sandpiper | Bird | Non-forest | NA | 839 | 0.92 | 0.97 | 0.95 |
| *Calypte anna* | Anna's hummingbird | Bird | Forest generalist | 4 | 8492 | 0.99 | 1.00 | 0.99 |
| *Cardellina pusilla* | Wilson's warbler | Bird | Forest generalist | 4 | 7695 | 0.80 | 0.94 | 0.86 |
| *Carduelis flammea* | Common redpoll | Bird | Forest generalist | 4 | 3052 | 0.86 | 0.91 | 0.95 |
| *Carduelis hornemanni* | Hoary redpoll | Bird | Non-forest | NA | 168 | 0.90 | 0.96 | 0.94 |
| *Carduelis pinus* | Pine siskin | Bird | Forest generalist | 4 | 28928 | 0.86 | 0.95 | 0.91 |
| *Carduelis tristis* | American goldfinch | Bird | Forest generalist | 4 | 21889 | 0.97 | 0.98 | 0.99 |
| *Carpodacus cassinii* | Cassin's finch | Bird | Forest generalist | 5 | 445 | 0.89 | 0.93 | 0.96 |
| *Carpodacus mexicanus* | House finch | Bird | Forest generalist | 5 | 2004 | 0.96 | 0.99 | 0.97 |
| *Carpodacus purpureus* | Purple finch | Bird | Forest generalist | 4 | 754 | 0.84 | 0.90 | 0.94 |
| *Cathartes aura* | Turkey vulture | Bird | Forest generalist | 4 | 7003 | 0.93 | 0.97 | 0.96 |
| *Catharus fuscescens* | Veery | Bird | Forest generalist | 4 | 2704 | 0.94 | 0.98 | 0.96 |
| *Catharus guttatus5* | Hermit thrush | Bird | Forest generalist | 5 | 4602 | 0.68 | 0.77 | 0.91 |
| *Catharus ustulatus* | Swainson's thrush | Bird | Non-forest | 3 | 9039 | 0.76 | 0.94 | 0.82 |
| *Catherpes mexicanus* | Canyon wren | Bird | Non-forest | 3 | 635 | 0.99 | 0.99 | 1.00 |
| *Cepphus columba* | Pigeon guillemot | Bird | Non-forest | 4 | 363 | 0.97 | 0.99 | 0.98 |
| *Cerorhinca monocerata* | Rhinoceros auklet | Bird | Non-forest | 4 | 198 | 0.95 | 0.97 | 0.98 |
| *Certhia americana* | Brown creeper | Bird | Forest specialist | 4 | 6238 | 0.89 | 0.96 | 0.93 |
| *Chaetura vauxi* | Vaux's swift | Bird | Forest specialist | 4 | 2664 | 0.90 | 0.94 | 0.96 |
| *Charadrius semipalmatus* | Semipalmated plover | Bird | Non-forest | 4 | 675 | 0.90 | 0.95 | 0.95 |
| *Charadrius vociferus* | Killdeer | Bird | Non-forest | 4 | 12317 | 0.92 | 0.95 | 0.97 |
| *Chen caerulescens* | Snow goose | Bird | Non-forest | 4 | 1103 | 0.96 | 0.99 | 0.97 |
| *Chlidonias niger* | Black tern | Bird | Non-forest | 4 | 695 | 0.89 | 0.97 | 0.92 |
| *Chondestes grammacus* | Lark sparrow | Bird | Non-forest | 2 | 473 | 0.97 | 0.99 | 0.98 |
| *Chordeiles minor* | Common nighthawk | Bird | Non-forest | 4 | 5472 | 0.83 | 0.91 | 0.92 |
| *Chroicocephalus philadelphia* | Bonaparte's gull | Bird | Forest generalist | 5 | 1850 | 0.88 | 0.93 | 0.95 |
| *Cinclus mexicanus* | American dipper | Bird | Forest generalist | 5 | 2332 | 0.87 | 0.95 | 0.92 |
| *Circus cyaneus* | Northern harrier | Bird | Non-forest | 4 | 6020 | 0.91 | 0.95 | 0.96 |
| *Cistothorus palustris* | Marsh wren | Bird | Non-forest | 5 | 8089 | 0.94 | 0.97 | 0.97 |
| *Clangula hyemalis* | Long-tailed duck | Bird | Non-forest | 2 | 861 | 0.89 | 0.92 | 0.97 |
| *Coccothraustes vespertinus* | Evening grosbeak | Bird | Forest generalist | 5 | 8603 | 0.87 | 0.96 | 0.91 |
| *Colaptes auratus* | Northern flicker | Bird | Forest generalist | 5 | 49736 | 0.89 | 0.92 | 0.97 |
| *Contopus cooperi* | Olive-sided flycatcher | Bird | Forest generalist | 3 | 2548 | 0.74 | 0.93 | 0.81 |
| *Contopus sordidulus* | Western wood pewee | Bird | Forest generalist | 4 | 7834 | 0.85 | 0.96 | 0.89 |
| *Corvus brachyrhynchos* | American crow | Bird | Forest generalist | 5 | 28918 | 0.89 | 0.97 | 0.92 |
| *Corvus caurinus* | Northwestern crow | Bird | Forest generalist | 5 | 27501 | 0.98 | 0.99 | 0.99 |
| *Corvus corax* | Common raven | Bird | Forest generalist | 5 | 32526 | 0.85 | 0.95 | 0.90 |
| *Cyanocitta cristata* | Blue-jay | Bird | Forest generalist | 4 | 762 | 0.93 | 0.98 | 0.95 |
| *Cyanocitta stelleri* | Steller's jay | Bird | Forest generalist | 5 | 22657 | 0.90 | 0.96 | 0.94 |
| *Cygnus buccinator* | Trumpeter swan | Bird | Non-forest | 4 | 4609 | 0.92 | 0.97 | 0.95 |
| *Cygnus columbianus* | Tundra swan | Bird | Non-forest | 3 | 1588 | 0.93 | 0.98 | 0.95 |
| *Cygnus olor* | Mute swan | Bird | Non-forest | NA | 689 | 0.99 | 1.00 | 0.99 |
| *Cypseloides niger* | American black swift | Bird | Non-forest | 4 | 1246 | 0.87 | 0.96 | 0.91 |
| *Dendragapus fuliginosus* | Sooty grouse | Bird | Forest generalist | 3 | 2781 | 0.85 | 0.94 | 0.91 |
| *Dendragapus obscurus* | Dusky grouse | Bird | Forest generalist | 4 | 1086 | 0.75 | 0.89 | 0.86 |
| *Dendroica petechia* | American yellow warbler | Bird | Forest generalist | 4 | 9901 | 0.86 | 0.93 | 0.93 |
| *Dendroica townsendi* | Townsend's warbler | Bird | Forest generalist | 5 | 5555 | 0.79 | 0.87 | 0.92 |
| *Dolichonyx oryzivorus* | Bobolink | Bird | Non-forest | 3 | 528 | 0.96 | 0.99 | 0.97 |
| *Dryocopus pileatus* | Pileated woodpecker | Bird | Forest specialist | 5 | 8512 | 0.88 | 0.93 | 0.95 |
| *Dumetella carolinensis* | Gray catbird | Bird | Non-forest | 5 | 5313 | 0.96 | 0.98 | 0.98 |
| *Empidonax alnorum* | Alder flycatcher | Bird | Forest generalist | 5 | 1785 | 0.79 | 0.95 | 0.84 |
| *Empidonax difficilis* | Pacific-slope flycatcher | Bird | Forest generalist | 4 | 4466 | 0.88 | 0.94 | 0.94 |
| *Empidonax flaviventris* | Yellow-bellied flycatcher | Bird | Forest generalist | 4 | 107 | 0.89 | 0.96 | 0.93 |
| *Empidonax hammondii* | Hammond's flycatcher | Bird | Forest generalist | 5 | 5836 | 0.80 | 0.90 | 0.90 |
| *Empidonax minimus* | Least flycatcher | Bird | Forest generalist | 5 | 1949 | 0.82 | 0.89 | 0.93 |
| *Empidonax oberholseri* | American dusky flycatcher | Bird | Forest generalist | 5 | 4198 | 0.83 | 0.96 | 0.87 |
| *Empidonax occidentalis* | Cordilleran flycatcher | Bird | Forest generalist | NA | 104 | 0.98 | 1.00 | 0.98 |
| *Empidonax traillii* | Willow flycatcher | Bird | Forest generalist | 4 | 6842 | 0.88 | 0.96 | 0.92 |
| *Empidonax wrightii* | Gray flycatcher | Bird | Forest specialist | 3 | 217 | 1.00 | 1.00 | 1.00 |
| *Eremophila alpestris* | Horned lark | Bird | Non-forest | 4 | 1086 | 0.84 | 0.95 | 0.89 |
| *Euphagus carolinus* | Rusty blackbird | Bird | Forest generalist | 3 | 524 | 0.74 | 0.89 | 0.85 |
| *Euphagus cyanocephalus* | Brewer's blackbird | Bird | Forest generalist | 5 | 12164 | 0.92 | 0.95 | 0.97 |
| *Falcipennis canadensis* | Spruce grouse | Bird | Forest generalist | 5 | 812 | 0.64 | 0.76 | 0.88 |
| *Falco columbarius* | Merlin | Bird | Forest generalist | 5 | 5286 | 0.89 | 0.93 | 0.96 |
| *Falco mexicanus* | Prairie falcon | Bird | Non-forest | 1 | 419 | 0.93 | 0.97 | 0.96 |
| *Falco peregrinus* | Peregrine falcon | Bird | Non-forest | 3 | 3955 | 0.92 | 0.99 | 0.93 |
| *Falco rusticolus* | Gyrfalcon | Bird | Non-forest | 3 | 403 | 0.92 | 0.98 | 0.94 |
| *Falco sparverius* | American kestrel | Bird | Forest generalist | 4 | 5930 | 0.87 | 0.92 | 0.95 |
| *Fulica americana* | American coot | Bird | Non-forest | 4 | 14948 | 0.94 | 0.97 | 0.97 |
| *Gallinago delicata* | Wilson's snipe | Bird | Non-forest | 4 | 5559 | 0.83 | 0.94 | 0.89 |
| *Gavia immer* | Common loon | Bird | Non-forest | 5 | 8910 | 0.82 | 0.95 | 0.87 |
| *Gavia pacifica* | Pacific loon | Bird | Non-forest | 4 | 489 | 0.90 | 0.93 | 0.97 |
| *Gavia stellata* | Red-throated loon | Bird | Non-forest | 4 | 421 | 0.95 | 0.99 | 0.96 |
| *Geothlypis tolmiei* | MacGillivray's warbler | Bird | Forest generalist | 4 | 4055 | 0.80 | 0.86 | 0.94 |
| *Geothlypis trichas* | Common yellowthroat | Bird | Non-forest | 5 | 11324 | 0.86 | 0.95 | 0.91 |
| *Glaucidium gnoma* | Northern pygmy owl | Bird | Forest generalist | 4 | 2838 | 0.81 | 0.88 | 0.93 |
| *Glaucomys sabrinus* | Northern flying squirrel | Bird | Forest generalist | 5 | 201 | 0.84 | 0.92 | 0.92 |
| *Grus canadensis* | Sandhill crane | Bird | Non-forest | 4 | 3442 | 0.83 | 0.94 | 0.89 |
| *Haematopus bachmani* | Black oystercatcher | Bird | Non-forest | 4 | 579 | 0.98 | 0.99 | 0.99 |
| *Haliaeetus leucocephalus* | Bald eagle | Bird | Forest generalist | 5 | 24167 | 0.91 | 0.94 | 0.97 |
| *Himantopus mexicanus* | Black-necked stilt | Bird | Non-forest | NA | 138 | 0.99 | 0.99 | 1.00 |
| *Hirundo rustica* | Barn swallow | Bird | Non-forest | 3 | 14314 | 0.89 | 0.96 | 0.93 |
| *Histrionicus histrionicus* | Harlequin duck | Bird | Non-forest | 3 | 1118 | 0.89 | 0.94 | 0.95 |
| *Hydroprogne caspia* | Caspian tern | Bird | Non-forest | 3 | 1067 | 0.98 | 0.99 | 0.99 |
| *Icteria virens* | Yellow-breasted chat | Bird | Forest generalist | 1 | 415 | 0.98 | 1.00 | 0.98 |
| *Icterus bullockii* | Bullock's oriole | Bird | Forest generalist | 5 | 4782 | 0.96 | 0.99 | 0.97 |
| *Icterus galbula* | Baltimore oriole | Bird | Forest generalist | 4 | 159 | 0.96 | 0.99 | 0.97 |
| *Ixoreus naevius* | Varied thrush | Bird | Forest generalist | 5 | 11164 | 0.83 | 0.94 | 0.89 |
| *Junco hyemalis* | Dark-eyed junco | Bird | Forest generalist | 5 | 44462 | 0.86 | 0.95 | 0.91 |
| *Lagopus lagopus* | Willow ptarmigan | Bird | Non-forest | 5 | 277 | 0.78 | 0.83 | 0.95 |
| *Lagopus leucura* | White-tailed ptarmigan | Bird | Non-forest | 5 | 314 | 0.75 | 0.88 | 0.87 |
| *Lagopus muta* | Rock ptarmigan | Bird | Non-forest | 5 | 100 | 0.90 | 0.95 | 0.95 |
| *Lanius excubitor* | Norhtern shrike | Bird | Forest generalist | 4 | 2209 | 0.91 | 0.95 | 0.96 |
| *Larus argentatus* | Herring gull | Bird | Non-forest | 4 | 5047 | 0.92 | 0.97 | 0.95 |
| *Larus californicus* | California gull | Bird | Non-forest | 3 | 8792 | 0.96 | 1.00 | 0.96 |
| *Larus canus* | Common gull | Bird | Non-forest | 5 | 4892 | 0.94 | 0.97 | 0.97 |
| *Larus delawarensis* | Ring-billed gull | Bird | Non-forest | 4 | 11515 | 0.96 | 0.99 | 0.97 |
| *Larus glaucescens* | Glaucous-winged gull | Bird | Non-forest | 5 | 18681 | 0.97 | 0.98 | 0.99 |
| *Larus hyperboreus* | Glaucous gull | Bird | Non-forest | NR | 266 | 0.97 | 0.99 | 0.98 |
| *Larus occidentalis* | Western gull | Bird | Non-forest | 4 | 288 | 0.98 | 1.00 | 0.98 |
| *Larus thayeri* | Thayer's gull | Bird | Non-forest | 5 | 1334 | 0.97 | 0.98 | 0.99 |
| *Leucophaeus pipixcan* | Fanklin's gull | Bird | Non-forest | 4 | 326 | 0.96 | 0.99 | 0.97 |
| *Leucosticte tephrocotis* | Gray-crowned rosy finch | Bird | Non-forest | 5 | 576 | 0.75 | 0.89 | 0.86 |
| *Limnodromus griseus* | Short-billed dowitcher | Bird | Non-forest | 2 | 353 | 0.92 | 0.97 | 0.95 |
| *Limnodromus scolopaceus* | Long-billed dowticher | Bird | Non-forest | 5 | 1983 | 0.94 | 0.97 | 0.97 |
| *Limosa haemastica* | Hudsonian godwit | Bird | Non-forest | 1 | 131 | 0.99 | 1.00 | 0.99 |
| *Lophodytes cucullatus* | Hooded merganser | Bird | Forest generalist | 5 | 9294 | 0.93 | 0.96 | 0.97 |
| *Loxia curvirostra* | Red crossbill | Bird | Forest generalist | 4 | 8114 | 0.84 | 0.93 | 0.91 |
| *Loxia leucoptera* | Two-barred crossbill | Bird | Forest generalist | 5 | 790 | 0.70 | 0.91 | 0.79 |
| *Megaceryle alcyon* | Belted kingfisher | Bird | Forest generalist | 4 | 10691 | 0.90 | 0.96 | 0.94 |
| *Megascops kennicottii* | Western screech-owl | Bird | Forest generalist | 4 | 414 | 0.93 | 0.98 | 0.95 |
| *Melanerpes lewis* | Lewis's woodpecker | Bird | Forest generalist | 2 | 1088 | 0.95 | 0.99 | 0.96 |
| *Melanitta fusca* | Velvet scooter | Bird | Non-forest | 4 | 803 | 0.89 | 0.94 | 0.95 |
| *Melanitta nigra* | Common scoter | Bird | Non-forest | 4 | 251 | 0.99 | 1.00 | 0.99 |
| *Melanitta perspicillata* | Surf scoter | Bird | Non-forest | 3 | 3771 | 0.90 | 0.97 | 0.93 |
| *Melospiza georgiana* | Swamp sparrow | Bird | Non-forest | 4 | 322 | 0.89 | 0.95 | 0.94 |
| *Melospiza lincolnii* | Lincoln's sparrow | Bird | Non-forest | 5 | 7424 | 0.79 | 0.85 | 0.94 |
| *Melospiza melodia* | Song sparrow | Bird | Non-forest | 5 | 49264 | 0.91 | 0.94 | 0.97 |
| *Mergus merganser* | Common merganser | Bird | Non-forest | 5 | 346 | 0.93 | 0.97 | 0.96 |
| *Mergus serrator* | Red-breasted merganser | Bird | Non-forest | 5 | 1324 | 0.92 | 0.96 | 0.96 |
| *Mniotilta varia* | Black and white warbler | Bird | Forest generalist | 4 | 161 | 0.95 | 0.98 | 0.97 |
| *Molothrus ater* | Brown-headed cowbird | Bird | Forest generalist | 5 | 10794 | 0.88 | 0.98 | 0.90 |
| *Myadestes townsendi* | Townsend's solitaire | Bird | Forest generalist | 4 | 3852 | 0.84 | 0.95 | 0.89 |
| *Nucifraga columbiana* | Clark's nutcracker | Bird | Forest generalist | 5 | 4939 | 0.88 | 0.93 | 0.95 |
| *Numenius americanus* | Long-billed curlew | Bird | Non-forest | 3 | 590 | 0.93 | 0.99 | 0.94 |
| *Numenius phaeopus* | Whimbrel | Bird | Non-forest | 4 | 115 | 0.97 | 0.99 | 0.98 |
| *Oreoscoptes montanus* | Sage thrasher | Bird | Non-forest | 1 | 109 | 0.99 | 0.99 | 1.00 |
| *Oreothlypis celata* | Orange-crowned warbler | Bird | Forest generalist | 5 | 12141 | 0.79 | 0.88 | 0.91 |
| *Oreothlypis peregrina* | Tennessee warbler | Bird | Forest generalist | 5 | 616 | 0.77 | 0.88 | 0.89 |
| *Oreothlypis ruficapilla* | Nashville warbler | Bird | Forest generalist | 5 | 3393 | 0.92 | 0.97 | 0.95 |
| *Otus flammeolus* | Flammulated owl | Bird | Forest specialist | 3 | 512 | 0.94 | 0.99 | 0.95 |
| *Oxyura jamaicensis* | Ruddy duck | Bird | Non-forest | 5 | 3708 | 0.92 | 0.96 | 0.96 |
| *Pandion haliaetus* | Osprey | Bird | Forest generalist | 5 | 12292 | 0.91 | 0.97 | 0.94 |
| *Parkesia noveboracensis* | Northern waterthrush | Bird | Forest generalist | 5 | 3749 | 0.77 | 0.86 | 0.91 |
| *Passerculus sandwichensis* | Savannah sparrow | Bird | Forest generalist | 4 | 9092 | 0.84 | 0.92 | 0.92 |
| *Passerella iliaca* | Fox sparrow | Bird | Forest generalist | 5 | 9451 | 0.88 | 0.94 | 0.94 |
| *Passerina amoena* | Lazuli bunting | Bird | Non-forest | 5 | 3187 | 0.93 | 0.98 | 0.95 |
| *Patagioenas fasciata* | Band-tailed pigeon | Bird | Forest generalist | 3 | 3011 | 0.96 | 0.99 | 0.97 |
| *Pelecanus erythrorhynchos* | American white pelican | Bird | Non-forest | 1 | 1973 | 0.96 | 0.99 | 0.97 |
| *Perisoreus canadensis* | Gray jay | Bird | Forest generalist | 5 | 3604 | 0.65 | 0.94 | 0.71 |
| *Petrochelidon pyrrhonota* | Cliff swallow | Bird | Non-forest | 4 | 4135 | 0.87 | 0.95 | 0.92 |
| *Phalacrocorax auritus* | Double-crested cormorant | Bird | Forest generalist | 3 | 7166 | 0.98 | 0.99 | 0.99 |
| *Phalacrocorax pelagicus* | Pelagic cormorant | Bird | Non-forest | 4 | 6100 | 0.98 | 0.99 | 0.99 |
| *Phalacrocorax penicillatus* | Brandt's cormorant | Bird | Non-forest | 4 | 285 | 0.97 | 0.98 | 0.99 |
| *Phalaenoptilus nuttallii* | Common poorwill | Bird | Non-forest | 4 | 642 | 0.95 | 0.98 | 0.97 |
| *Phalaropus lobatus* | Red-necked pharalope | Bird | Non-forest | 3 | 1045 | 0.87 | 0.91 | 0.96 |
| *Phalaropus tricolor* | Wilson's pharalope | Bird | Non-forest | 4 | 972 | 0.90 | 0.94 | 0.96 |
| *Pheucticus ludovicianus* | Rose-breasted grosbreak | Bird | Forest generalist | 4 | 283 | 0.94 | 0.98 | 0.96 |
| *Pheucticus melanocephalus* | Black-headed grosbeak | Bird | Forest generalist | 5 | 6735 | 0.93 | 0.98 | 0.95 |
| *Pica hudsonia* | Black-billed magpie | Bird | Forest generalist | 5 | 14617 | 0.94 | 0.99 | 0.95 |
| *Picoides arcticus* | Black-backed woodpecker | Bird | Forest generalist | 5 | 311 | 0.75 | 0.86 | 0.89 |
| *Picoides dorsalis* | American three-toed woodpecker | Bird | Forest specialist | 5 | 821 | 0.71 | 0.85 | 0.86 |
| *Picoides pubescens* | Downy woodpecker | Bird | Forest generalist | 5 | 19231 | 0.93 | 0.95 | 0.98 |
| *Picoides villosus* | Hairy woodpecker | Bird | Forest generalist | 5 | 9792 | 0.85 | 0.89 | 0.96 |
| *Pinicola enucleator* | Pine grosbeak | Bird | Forest generalist | 5 | 2741 | 0.77 | 0.84 | 0.93 |
| *Pipilo maculatus* | Spotted towhee | Bird | Forest generalist | 5 | 31677 | 0.95 | 0.97 | 0.98 |
| *Piranga ludoviciana* | Western tanager | Bird | Forest generalist | 5 | 10331 | 0.83 | 0.89 | 0.94 |
| *Plectrophenax nivalis* | Snow bunting | Bird | Non-forest | 4 | 344 | 0.86 | 0.95 | 0.91 |
| *Pluvialis dominica* | American golden plover | Bird | Non-forest | 3 | 333 | 0.93 | 0.98 | 0.95 |
| *Pluvialis squatarola* | Black-bellied plover | Bird | Non-forest | 5 | 678 | 0.98 | 0.99 | 0.99 |
| *Podiceps auritus* | Horned grebe | Bird | Non-forest | 4 | 9703 | 0.91 | 0.96 | 0.95 |
| *Podiceps grisegena* | Red-necked grebe | Bird | Non-forest | 4 | 4249 | 0.87 | 0.95 | 0.92 |
| *Podiceps nigricollis* | Eared grebe | Bird | Non-forest | 4 | 808 | 0.91 | 0.97 | 0.94 |
| *Podilymbus podiceps* | Pie-billed grebe | Bird | Non-forest | 4 | 8539 | 0.95 | 0.98 | 0.97 |
| *Poecile atricapillus* | Black-capped chickadee | Bird | Forest generalist | 5 | 49608 | 0.91 | 0.99 | 0.92 |
| *Poecile gambeli* | Mountain chickadee | Bird | Forest generalist | 5 | 14557 | 0.87 | 0.96 | 0.91 |
| *Poecile hudsonica* | Boreal chickadee | Bird | Forest generalist | 5 | 695 | 0.63 | 0.82 | 0.81 |
| *Poecile rufescens* | Chestnut-backed chickadee | Bird | Forest generalist | 4 | 15137 | 0.93 | 0.96 | 0.97 |
| *Pooecetes gramineus* | Vesper sparrow | Bird | Non-forest | 4 | 2618 | 0.91 | 0.96 | 0.95 |
| *Porzana carolina* | Sora | Bird | Non-forest | 4 | 2554 | 0.89 | 0.96 | 0.93 |
| *Progne subis* | Purple martin | Bird | Forest generalist | 2 | 1781 | 0.99 | 0.99 | 1.00 |
| *Psaltriparus minimus* | American bushtit | Bird | Forest generalist | 5 | 8277 | 0.99 | 0.99 | 1.00 |
| *Quiscalus quiscula* | Common grackle | Bird | Forest generalist | 5 | 168 | 0.97 | 0.99 | 0.98 |
| *Rallus limicola* | Virginia rail | Bird | Non-forest | 4 | 2750 | 0.96 | 0.98 | 0.98 |
| *Recurvirostra americana* | American avocet | Bird | Non-forest | 2 | 826 | 0.97 | 0.98 | 0.99 |
| *Regulus calendula* | Ruby-crowned kinglet | Bird | Forest specialist | 5 | 15364 | 0.80 | 0.89 | 0.91 |
| *Regulus satrapa* | Golden-crowned kinglet | Bird | Forest specialist | 5 | 13506 | 0.81 | 0.88 | 0.93 |
| *Riparia riparia* | Sand martin | Bird | Non-forest | 4 | 2251 | 0.91 | 0.95 | 0.96 |
| *Salpinctes obsoletus* | Rock wren | Bird | Non-forest | 4 | 527 | 0.95 | 0.97 | 0.98 |
| *Sayornis phoebe* | Eastern phoebe | Bird | Forest generalist | 4 | 164 | 0.94 | 0.96 | 0.98 |
| *Sayornis saya* | Say's phoebe | Bird | Non-forest | 5 | 2280 | 0.93 | 0.97 | 0.96 |
| *Seiurus aurocapilla* | Ovenbird | Bird | Forest generalist | 4 | 156 | 0.90 | 0.97 | 0.93 |
| *Seiurus noveboracensis* | Northern waterthrush | Bird | Forest generalist | 5 | 111 | 0.87 | 0.94 | 0.93 |
| *Selasphorus calliope* | Calliope hummingbird | Bird | Forest generalist | 5 | 116 | 0.95 | 1.00 | 0.95 |
| *Selasphorus rufus* | Rufous hummingbird | Bird | Forest generalist | 4 | 14005 | 0.87 | 0.96 | 0.91 |
| *Setophaga auduboni* | Audobon's warbler | Bird | Forest generalist | NA | 3634 | 0.83 | 0.89 | 0.94 |
| *Setophaga coronata* | Yellow-rumped warbler | Bird | Forest generalist | 5 | 20979 | 0.82 | 0.94 | 0.88 |
| *Setophaga magnolia* | Magnolia warbler | Bird | Forest generalist | 4 | 1240 | 0.86 | 0.94 | 0.92 |
| *Setophaga nigrescens* | Black-throated gray warbler | Bird | Forest generalist | 4 | 2095 | 0.97 | 0.99 | 0.98 |
| *Setophaga ruticilla* | American redstart | Bird | Forest generalist | 5 | 3997 | 0.90 | 0.92 | 0.98 |
| *Setophaga striata* | Blackpoll warbler | Bird | Forest generalist | 5 | 256 | 0.75 | 0.84 | 0.91 |
| *Sialia currucoides* | Mountain bluebird | Bird | Non-forest | 4 | 3347 | 0.87 | 0.92 | 0.95 |
| *Sialia mexicana* | Western bluebird | Bird | Forest generalist | 4 | 2299 | 0.96 | 0.98 | 0.98 |
| *Sitta canadensis* | Red-breasted nuthatch | Bird | Forest generalist | 5 | 31230 | 0.86 | 0.90 | 0.96 |
| *Sitta carolinensis* | White-breasted nuthatch | Bird | Forest generalist | 5 | 2328 | 0.94 | 0.98 | 0.96 |
| *Sitta pygmaea* | Pygmy nuthatch | Bird | Forest specialist | 4 | 4784 | 0.98 | 1.00 | 0.98 |
| *Sphyrapicus nuchalis* | Red-naped sapsucker | Bird | Forest specialist | 5 | 4417 | 0.90 | 0.97 | 0.93 |
| *Sphyrapicus ruber* | Red-breasted sapsucker | Bird | Forest generalist | 5 | 2553 | 0.86 | 0.92 | 0.94 |
| *Sphyrapicus thyroideus* | Williamson's sapsucker | Bird | Forest generalist | 3 | 240 | 0.97 | 0.99 | 0.98 |
| *Sphyrapicus varius* | Yellow-bellied sapsucker | Bird | Forest generalist | 5 | 465 | 0.80 | 0.88 | 0.92 |
| *Spizella arborea* | American tree sparrow | Bird | Forest generalist | 5 | 768 | 0.86 | 0.93 | 0.93 |
| *Spizella breweri* | Brewer's sparrow | Bird | Non-forest | 4 | 420 | 0.92 | 0.97 | 0.95 |
| *Spizella pallida* | Clay-colored sparrow | Bird | Forest generalist | 4 | 1029 | 0.89 | 0.94 | 0.95 |
| *Spizella passerina* | Chipping sparrow | Bird | Forest generalist | 5 | 11886 | 0.79 | 0.94 | 0.85 |
| *Stelgidopteryx serripennis* | Northern rough-winged swallow | Bird | Non-forest | 4 | 6296 | 0.90 | 0.95 | 0.95 |
| *Stercorarius parasiticus* | Parasitic jaeger | Bird | Non-forest | 1 | 124 | 0.93 | 0.96 | 0.97 |
| *Sterna forsteri* | Foster's tern | Bird | Non-forest | 1 | 139 | 0.99 | 1.00 | 0.99 |
| *Sterna hirundo* | Common tern | Bird | Non-forest | 4 | 295 | 0.94 | 0.98 | 0.96 |
| *Sterna paradisaea* | Arctic tern | Bird | Non-forest | 4 | 110 | 0.88 | 0.95 | 0.93 |
| *Strix nebulosa* | Great gray owl | Bird | Forest specialist | 4 | 141 | 0.78 | 0.94 | 0.84 |
| *Strix varia* | Barred owl | Bird | Forest generalist | 5 | 1594 | 0.89 | 0.93 | 0.96 |
| *Sturnella neglecta* | Western meadowlark | Bird | Non-forest | 4 | 6653 | 0.93 | 0.97 | 0.96 |
| *Surnia ulula* | Northern hawk-owl | Bird | Forest generalist | 4 | 260 | 0.75 | 0.90 | 0.85 |
| *Synthliboramphus antiquus* | Ancient murrelet | Bird | Forest generalist | 2 | 127 | 0.96 | 0.98 | 0.98 |
| *Tachycineta bicolor* | Tree swallow | Bird | Forest generalist | 4 | 15622 | 0.87 | 0.92 | 0.95 |
| *Tachycineta thalassina* | Violet-green swallow | Bird | Forest generalist | 4 | 17244 | 0.91 | 0.94 | 0.97 |
| *Thryomanes bewickii* | Bewick's wren | Bird | Forest generalist | 4 | 7620 | 1.00 | 1.00 | 1.00 |
| *Tringa flavipes* | Lesser yellowlegs | Bird | Non-forest | 5 | 2030 | 0.89 | 0.92 | 0.97 |
| *Tringa incana* | Wandering tattler | Bird | Non-forest | 3 | 107 | 0.90 | 0.97 | 0.93 |
| *Tringa melanoleuca* | Greater yellowlegs | Bird | Non-forest | 5 | 3740 | 0.90 | 0.92 | 0.98 |
| *Tringa solitaria* | Solitary sandpiper | Bird | Non-forest | 5 | 1249 | 0.80 | 0.94 | 0.86 |
| *Troglodytes aedon* | House wren | Bird | Forest generalist | 5 | 2488 | 0.94 | 0.98 | 0.96 |
| *Troglodytes hiemalis* | Winter wren | Bird | Forest specialist | 5 | 11092 | 0.89 | 0.95 | 0.94 |
| *Troglodytes pacificus* | Pacific wren | Bird | Forest generalist | 5 | 9655 | 0.87 | 0.96 | 0.91 |
| *Turdus migratorius* | American robin | Bird | Forest generalist | 5 | 67199 | 0.85 | 0.96 | 0.89 |
| *Tympanuchus phasianellus* | Sharp-tailed grouse | Bird | Non-forest | 4 | 238 | 0.90 | 0.95 | 0.95 |
| *Tyrannus tyrannus* | Eastern kingbird | Bird | Forest generalist | 4 | 4683 | 0.92 | 0.96 | 0.96 |
| *Tyrannus verticalis* | Western kingbird | Bird | Forest generalist | 4 | 2796 | 0.95 | 0.99 | 0.96 |
| *Tyto alba* | Barn owl | Bird | Non-forest | 3 | 629 | 0.98 | 0.99 | 0.99 |
| *Uria aalge* | Common murre | Bird | Non-forest | 2 | 390 | 0.97 | 0.99 | 0.98 |
| *Vireo cassinii* | Cassin's vireo | Bird | Forest generalist | 5 | 3497 | 0.86 | 0.93 | 0.93 |
| *Vireo gilvus* | Warbling vireo | Bird | Forest generalist | 5 | 14881 | 0.78 | 0.95 | 0.83 |
| *Vireo huttoni* | Hutton's vireo | Bird | Forest generalist | 4 | 853 | 0.98 | 1.00 | 0.98 |
| *Vireo olivaceus* | Red-eyed vireo | Bird | Forest generalist | 4 | 4540 | 0.88 | 0.96 | 0.92 |
| *Vireo solitarius* | Blue-headed vireo | Bird | Forest generalist | 4 | 312 | 0.81 | 0.89 | 0.92 |
| *Xanthocephalus xanthocephalus* | Yellow-headed blackbird | Bird | Non-forest | 4 | 4636 | 0.94 | 0.98 | 0.96 |
| *Zenaida macroura* | Mourning dove | Bird | Forest generalist | 4 | 6860 | 0.95 | 0.99 | 0.96 |
| *Zonotrichia albicollis* | White-throated sparrow | Bird | Forest generalist | 5 | 2355 | 0.86 | 0.94 | 0.92 |
| *Zonotrichia atricapilla* | Golden-crowned sparrow | Bird | Non-forest | 5 | 7701 | 0.92 | 0.95 | 0.97 |
| *Zonotrichia leucophrys* | White-crowned sparrow | Bird | Forest generalist | 5 | 15794 | 0.87 | 0.96 | 0.91 |
| *Zonotrichia querula* | Harris's sparrow | Bird | Forest generalist | NA | 174 | 0.95 | 0.99 | 0.96 |
| *Achrocheilus alutaceus* | Chiselmouth | Fish | Aquatic | 3 | - | - | - | - |
| *Acipenser transmontanus* | White sturgeon | Fish | Aquatic | 2 | - | - | - | - |
| *Catostomus catostomus* | Longnose sucker | Fish | Aquatic | 5 | - | - | - | - |
| *Catostomus columbianus* | Bridgelip sucker | Fish | Aquatic | 5 | - | - | - | - |
| *Catostomus commersonii* | White sucker | Fish | Aquatic | 5 | - | - | - | - |
| *Catostomus macrocheilus* | Largescale sucker | Fish | Aquatic | 5 | - | - | - | - |
| *Coregonus clupeaformis* | Lake whitefish | Fish | Aquatic | 5 | - | - | - | - |
| *Cottus aleuticus* | Coastrange sculpin | Fish | Aquatic | 5 | - | - | - | - |
| *Cottus asper* | Prickly sculpin | Fish | Aquatic | 5 | - | - | - | - |
| *Cottus cognatus* | Slimy sculpin | Fish | Aquatic | 5 | - | - | - | - |
| *Couesius plumbeus* | Lake chub | Fish | Aquatic | 5 | - | - | - | - |
| *Esox lucius* | Northern pike | Fish | Aquatic | 5 | - | - | - | - |
| *Gasterosteus aculeatus* | Threespine stickleback | Fish | Aquatic | 5 | - | - | - | - |
| *Lota lota* | Burbot | Fish | Aquatic | 4 | - | - | - | - |
| *Mylocheilus caurinus* | Peamouth chub | Fish | Aquatic | 5 | - | - | - | - |
| *Oncorhynchus clarkia clarkii* | Coastal cutthroat trout | Fish | Aquatic | 3 | - | - | - | - |
| *Oncorhynchus clarkia lewisi* | Westslope cutthroat trout | Fish | Aquatic | 3 | - | - | - | - |
| *Oncorhynchus mykiss* | Rainbow trout | Fish | Aquatic | 5 | - | - | - | - |
| *Oncorhynchus nerka* | Kokanee | Fish | Aquatic | 4 | - | - | - | - |
| *Percopsis omiscomaycus* | Trout-perch | Fish | Aquatic | 4 | - | - | - | - |
| *Prosopium coulterii* | Pygmy whitefish | Fish | Aquatic | 4 | - | - | - | - |
| *Prosopium williamsoni* | Mountain whitefish | Fish | Aquatic | 5 | - | - | - | - |
| *Ptychocheilus oregonensis* | Northern pikeminnow | Fish | Aquatic | 5 | - | - | - | - |
| *Rhinichthys cataractae* | Longnose dace | Fish | Aquatic | 5 | - | - | - | - |
| *Rhinichtys falcatus* | Leopard dace | Fish | Aquatic | 4 | - | - | - | - |
| *Richardsonius balteatus* | Redside shiner | Fish | Aquatic | 5 | - | - | - | - |
| *Salvelinus confluentus* | Bull trout | Fish | Aquatic | 3 | - | - | - | - |
| *Salvelinus malma* | Dolly varden | Fish | Aquatic | 4 | - | - | - | - |
| *Salvelinus namaycush* | Lake trout | Fish | Aquatic | 4 | - | - | - | - |
| *Sander vitreus* | Walleye | Fish | Aquatic | 4 | - | - | - | - |
| *Thymallus arcticus* | Arctic grayling | Fish | Aquatic | 5 | - | - | - | - |
| *Ambystoma gracile* | Northwestern salamander | Herpetile | Forest generalist | 4 | 158 | 0.98 | 0.99 | 0.99 |
| *Ambystoma macrodactylum* | Long-toed salamander | Herpetile | Forest generalist | 4 | 217 | 0.94 | 0.98 | 0.96 |
| *Anaxyrus boreas* | Western toad | Herpetile | Forest generalist | 3 | 734 | 0.73 | 0.85 | 0.88 |
| *Aneides vagrans* | Wandering salamander | Herpetile | Forest generalist | 3 | 103 | 1.00 | 1.00 | 1.00 |
| *Ascaphus truei* | Coastal Tailed Frog | Herpetile | Forest specialist | 3 | 1922 | 0.88 | 0.95 | 0.93 |
| *Lithobates sylvaticus* | Wood frog | Herpetile | Forest generalist | 4 | 139 | 0.80 | 0.88 | 0.92 |
| *Plethodon vehiculum* | Western redback salamander | Herpetile | Forest generalist | 4 | 117 | 0.98 | 0.99 | 0.99 |
| *Pseudacris regilla* | Pacific tree frog | Herpetile | Forest generalist | 5 | 275 | 0.92 | 0.96 | 0.96 |
| *Rana aurora* | Northern red-legged frog | Herpetile | Forest generalist | 3 | 225 | 0.97 | 0.98 | 0.99 |
| *Rana luteiventris* | Columbia spotted frog | Herpetile | Non-forest | 4 | 355 | 0.79 | 0.90 | 0.89 |
| *Spea hammondii* | Western spadefoot | Herpetile | Non-forest | NA | 172 | 1.00 | 1.00 | 1.00 |
| *Taricha granulosa* | Rough-skinned newt | Herpetile | Forest generalist | 4 | 778 | 0.93 | 0.96 | 0.97 |
| *Thamnophis elegans* | Western garter snake | Herpetile | Forest generalist | 5 | 127 | 0.90 | 0.94 | 0.96 |
| *Thamnophis ordinoides* | NW garter snake | Herpetile | Non-forest | 4 | 110 | 0.99 | 1.00 | 0.99 |
| *Thamnophis sirtalis* | Common garter snake | Herpetile | Forest generalist | 5 | 157 | 0.83 | 0.94 | 0.89 |
| *Lemmus sibiricus* | Siberian brown lemming | Mammal | Non-forest | 5 | 156 | 0.96 | 1.00 | 0.96 |
| *Lepus americanus* | Snowshoe hare | Mammal | Forest generalist | 5 | 199 | 0.91 | 0.93 | 0.98 |
| *Martes americana* | American marten | Mammal | Forest specialist | 4 | 707 | 0.82 | 0.86 | 0.96 |
| *Martes pennanti* | Fisher | Mammal | Forest generalist | 2 | 140 | 0.88 | 0.94 | 0.94 |
| *Microtus longicaudus* | Long-tailed vole | Mammal | Forest generalist | 5 | 509 | 0.83 | 0.87 | 0.96 |
| *Microtus pennsylvanicus* | Meadow vole | Mammal | Non-forest | 5 | 975 | 0.81 | 0.85 | 0.96 |
| *Microtus townsendii* | Townsend's vole | Mammal | Non-forest | 5 | 267 | 0.98 | 0.99 | 0.99 |
| *Mustela erminea* | Stoat | Mammal | Forest generalist | 5 | 764 | 0.81 | 0.88 | 0.93 |
| *Myodes gapperi* | Southern red-backed vole | Mammal | Forest generalist | 5 | 789 | 0.85 | 0.94 | 0.91 |
| *Myodes rutilus* | Northern red-backed vole | Mammal | Forest generalist | 5 | 584 | 0.97 | 0.99 | 0.98 |
| *Myotis lucifugus* | Little brown bat | Mammal | Forest generalist | 4 | 181 | 0.96 | 0.97 | 0.99 |
| *Neotoma cinerea* | Bushy-tailed woodrat | Mammal | Forest generalist | 5 | 119 | 0.88 | 0.95 | 0.93 |
| *Neovison vison* | American mink | Mammal | Forest generalist | 5 | 350 | 0.82 | 0.86 | 0.96 |
| *Ochotona princeps* | American pika | Mammal | Forest specialist | 5 | 437 | 0.92 | 0.94 | 0.98 |
| *Peromyscus keeni* | Northwestern deer mouse | Mammal | Forest specialist | 5 | 591 | 0.88 | 0.93 | 0.95 |
| *Peromyscus maniculatus* | Deer mouse | Mammal | Forest generalist | 5 | 2722 | 0.78 | 0.87 | 0.91 |
| *Phenacomys intermedius* | Western heather vole | Mammal | Forest generalist | 5 | 149 | 0.86 | 0.96 | 0.90 |
| *Sorex cinereus* | Cinereus shrew | Mammal | Forest generalist | 5 | 617 | 0.87 | 0.91 | 0.96 |
| *Sorex monticolus* | Montane shrew | Mammal | Forest generalist | 5 | 714 | 0.79 | 0.88 | 0.91 |
| *Sorex vagrans* | Vagrant shrew | Mammal | Forest generalist | 5 | 896 | 0.96 | 0.99 | 0.97 |
| *Tamias amoenus* | Yellow-pine chipmunk | Mammal | Forest generalist | 5 | 419 | 0.84 | 0.90 | 0.94 |
| *Tamias minimus* | Least chipmunk | Mammal | Forest generalist | 5 | 105 | 0.89 | 0.91 | 0.98 |
| *Tamiasciurus hudsonicus* | American red squirrel | Mammal | Forest generalist | 5 | 651 | 0.77 | 0.87 | 0.90 |
| *Thomomys talpoides* | Northern pocket gopher | Mammal | Non-forest | 5 | 405 | 0.95 | 0.98 | 0.97 |
| *Zapus princeps* | Western jumping mouse | Mammal | Non-forest | 5 | 235 | 0.85 | 0.90 | 0.95 |
|  |  | **Taxon** |  |  | $\bar{\boldsymbol{n}}$ | $\bar{\mathbf{TSS}}$ | **TSS_max_** | **TSS_min_** |
|  |  | Mammal |  |  | 547.2 | 0.87 | 0.98 | 0.77 |
|  |  | Bird |  |  | 6005 | 0.90 | 1.0 | 0.63 |
|  |  | Herpeto fauna |  |  | 372.6 | 0.91 | 1.0 | 0.73 |
|  |  |  |  |  |  |  |  |  |
|  |  |  |  |  |  |  |  |  |
|  |  |  |  |  |  |  |  |  |

**Supporting Information S2.** Ensemble species distribution modeling methodology.

For modeling terrestrial vertebrate species distributions, we used a set of bioclimatic and topographic variables. We extracted bioclimatic data (annual precipitation, isothermality (mean diurnal range/annual temperature range), precipitation seasonality (coefficient of variation of monthly precipitation), temperature seasonality, mean temperature of the coldest quarter, and mean temperature of the warmest quarter) from the WorldClim database ([*www.worldclim.org*](http://www.worldclim.org); ^1^).We also used elevation data (225-m resolution) from USGS Global Multi-resolution Terrain Elevation Data model (*topotools.cr.usgs.gov/gmted_viewer*), and slope derived from the DEM using ArcGIS 10 (ESRI, Redlands, CA).

Distribution models were created using BIOMOD2 package ^2^ in program R. All inputs, and therefore all resulting outputs, were standardized to 1 km^2^ pixel size. Since we were limited to presence-only data, we chose pseudo-absences for each species at random from within a polygon constructed by aggregating 250 km radius polygons centered on every occurrence point. We selected this maximum pseudo-absence radius as a balance between incorrectly inflating the predicted probability of occurrence within the species range by sampling pseudo-absences from far outside of species distributions, and broad climatic trends may overshadow important ecological relationships in the outputs ^3,4^. We set the number of random pseudo-absences equal to the number of presences for each species (i.e. prevalence = 0.5) in order to avoid spurious measures of accuracy often encountered when prevalence is extremely high or low ^5,6^, then performed 3 pseudo-absence selection trials, each of which was modelled 8 replicate times using an 80/20 split for training/testing data ^7^. Ensemble models were built by testing a suite of 7 modelling techniques available in BIOMOD: generalized linear models, generalized additive models, classification tree analyses, artificial neural networks, surface range envelopes (BIOCLIM), multiple adaptive regression splines, and random forests. We used the true skill statistic (TSS; ^6^) to assess the accuracy of our models because it doesn’t suffer from the limitations of several other analytical statistics commonly used to assess the accuracy of SDMs ^6,8^. The TSS is also an easy metric to interpret: it is simply the sum of a model’s specificity (probability of a true positive) and its sensitivity (probability of a true negative) minus 1, and has a range of -1 to 1. We chose to include only individual models with a TSS higher than 0.3 in our final ensemble model for each species, and then delineated the final ensemble model into a binary presence-absence map by using the prevalence as the delineation threshold ^4^. Ensemble models are built in BIOMOD by converting every model output into a binary map, then taking the average for each cell across all models. Values near 0 or 1 in the ensemble can be interpreted as good model agreement on absence or presence of a species, and values near 0.5 indicate poor model agreement. Ensemble models of forest specialist species were clipped post-hoc to exclude areas of urban, barren, and agricultural land cover using the Land Cover dataset provided by the Commission for Environmental Cooperation.

**Model performance** – All species were modelled with a high enough accuracy to retain for further analysis (mean TSS = 0.9, max = 1.0, min = 0.63). Despite having the lowest average number of input points, reptiles and amphibians were modelled best, closely followed by birds and mammals ($\bar{TSS}$ = 0.91, 0.90, and 0.87 respectively; Supporting Information S1).

**References**

1. Hijmans, R. J., Cameron, S. E., Parra, J. L., Jones, P. G. & Jarvis, A. Very high resolution interpolated climate surfaces for global land areas. *Int. J. Climatol.* **25**, 1965–1978 (2005).

2. Thuiller, W., Lafourcade, B., Engler, R. & Araujo, M. B. BIOMOD - a platform for ensemble forecasting of species distributions. *Ecography (Cop.).* **32**, 369–373 (2009).

3. VanDerWal, J., Shoo, L. P., Graham, C. & Williams, S. E. Selecting pseudo-absence data for presence-only distribution modeling: How far should you stray from what you know? *Ecol. Modell.* **220**, 589–594 (2009).

4. Stokland, J. N., Halvorsen, R. & Støa, B. Species distribution modelling—Effect of design and sample size of pseudo-absence observations. *Ecol. Modell.* **222**, 1800–1809 (2011).

5. McPhersonn, J. M., Jetz, W. & Rogers, D. J. The effects of species’ range sizes on the accuracy of distribution models: ecological phenomenon or statistical artefact? *J. Appl. Ecol.* **41**, 811–823 (2004).

6. Allouche, O., Tsoar, A. & Kadmon, R. Assessing the accuracy of species distribution models: prevalence, kappa and the true skill statistic (TSS). *J. Appl. Ecol.* **43**, 1223–1232 (2006).

7. Barbet-Massin, M., Jiguet, F., Albert, C. H. & Thuiller, W. Selecting pseudo-absences for species distribution models: how, where and how many? *Methods Ecol. Evol.* **3**, 327–338 (2012).

8. Lobo, J. M., Jiménez-Valverde, A. & Real, R. AUC: a misleading measure of the performance of predictive distribution models. *Glob. Ecol. Biogeogr.* **17**, 145–151 (2008).
